# Multi-Omics Integration Identifies a Six-Gene Diagnostic Signature for Ankylosing Spondylitis via Metabolic–Immune Crosstalk

**DOI:** 10.3390/ijms27093860

**Published:** 2026-04-27

**Authors:** Xuejian Dan, Xiangyuan Guan, Hangjian Hu, Wei Liu, Zhourui Wu, Xiao Hu, Wei Xu, Yunfei Zhao, Bin Ma

**Affiliations:** 1 Department of Orthopedics, Tongji Hospital, School of Medicine, Tongji University, Shanghai 200065, China; 2511305@tongji.edu.cn (X.D.); 2152997@tongji.edu.cn (H.H.); liuwei120120@163.com (W.L.); 2012wuzhourui@tongji.edu.cn (Z.W.); xueshenhuxiao@163.com (X.H.); david_xuwei@tongji.edu.cn (W.X.); zhaoyunfei9336@163.com (Y.Z.); 2 School of Computer Science and Engineering, Beihang University, Beijing 100191, China; gxy0615@buaa.edu.cn

**Keywords:** ankylosing spondylitis, metabolic reprogramming, machine learning, immune microenvironment, single-cell transcriptomics, ceRNA network, natural compounds

## Abstract

Ankylosing spondylitis (AS) is a chronic immune-mediated inflammatory disease affecting the axial skeleton, characterized by progressive structural damage and functional impairment. Although biologic therapies targeting tumor necrosis factor and interleukin-17 have improved clinical outcomes, a substantial proportion of patients fail to achieve sustained disease control. Emerging evidence suggests that metabolic alterations may contribute to AS pathogenesis; however, systematic characterization of metabolism-related biomarkers and their regulatory networks remains limited, and the interplay between metabolic dysfunction and immune dysregulation in AS is poorly understood. Two whole-blood GEO datasets (GSE25101, GSE73754; *n* = 104) were integrated as the primary analytical cohort. A third dataset (GSE11886, *n* = 18; monocyte-derived macrophages) was included for exploratory cross-tissue analysis. Differential expression analysis identified 847 DEGs, which were refined to 16 metabolism-related genes through weighted gene co-expression network analysis (WGCNA) and GeneCards database filtering. Eleven machine learning algorithms with 5-fold cross-validation were applied to construct diagnostic models and identify hub genes. Validation analyses included immune cell infiltration estimation using CIBERSORT, metabolic pathway activity assessment via ssGSEA, single-cell transcriptomics from GSE268839, functional enrichment through GSEA/GSVA, and chromosomal localization analysis. A competing endogenous RNA (ceRNA) regulatory network was constructed to map post-transcriptional regulation. Natural compounds from 66 AS-treating traditional Chinese medicines were screened against hub genes using deep learning-based binding prediction. Multiple machine learning algorithms achieved comparable cross-validated performance (CV AUC range 0.741–0.836; top five models: 0.805–0.836) using the six hub genes (*MFN2*, *SLC27A3*, *RHOB*, *SMG7*, *AKR1B1*, *LCOR*) identified through SHAP-based feature importance analysis of the PLS model. Leave-one-dataset-out validation between the two whole-blood cohorts showed that all algorithms exceeded an AUC of 0.77 in Round 1 (validate: GSE73754, *n* = 72; best AUC 0.861), while Round 2 (validate: GSE25101, *n* = 32) yielded more modest performance (best AUC, 0.715) reflecting the smaller validation sample. Exploratory application to GSE11886 (macrophage-derived samples) showed near-chance performance, consistent with the tissue-source discrepancy. AS patients exhibited significant downregulation of oxidative phosphorylation, TCA cycle, and glycolysis pathways (*p* < 0.01), accompanied by elevated glutathione metabolism (*p* < 0.001). Immune cell deconvolution revealed reduced CD8+ T cell proportions correlating with *MFN2* downregulation, and increased neutrophil frequencies correlating with *SLC27A3* upregulation. Exploratory single-cell analysis indicated that *RHOB* expression was relatively enriched in border-associated macrophages and fibroblasts, while *AKR1B1* was more prominently expressed in vascular endothelial cells and plasmacytoid dendritic cells. The ceRNA network identified 21 miRNAs and 65 lncRNAs forming 86 regulatory interactions, with four key regulatory axes (SATB1-AS1/miR-539-5p/*LCOR*, FAM95B1/miR-223-3p/*RHOB*, LINC01106/miR-106a-5p/*MFN2*, AATBC/miR-185-5p/*SMG7*) predicted to regulate hub gene expression. Compound screening identified betaine, pyruvic acid, citric acid, etc., as top-ranking candidates, with *MFN2* showing the highest binding capacity among hub genes. This study provides an integrative framework linking metabolic reprogramming with immune dysfunction in AS. The six-gene diagnostic signature showed preliminary discriminatory ability in the available datasets, while the ceRNA regulatory network and natural compound screening results prioritize candidate regulatory pathways and compounds for future validation. These findings advance our understanding of AS pathogenesis and may guide future biomarker development and targeted intervention strategies.

## 1. Introduction

Ankylosing spondylitis (AS) is a chronic immune-mediated inflammatory disease that predominantly affects the axial skeleton, which typically develops in early adulthood and is associated with persistent pain, progressive structural damage, and functional impairment [1]. Although biologic therapies targeting tumor necrosis factor and interleukin-17 (IL-17) have improved clinical outcome, a substantial proportion of patients fail to achieve sustained disease control [2]. Variable treatment response and long-term disease progression indicate that the pathogenic mechanisms of AS are not yet fully understood, and additional molecular insights are required to support improved therapeutic strategies.

Immune dysregulation is a key feature of AS pathogenesis. Previous studies have implicated the IL-23/IL-17 axis, innate immune activation, and altered interactions among immune cell populations [3]. However, immune abnormalities alone do not fully explain the variability in disease manifestations or therapeutic responses. Recently, immunometabolism has emerged as an important area of research, emphasizing the role of cellular metabolic processes in shaping immune cell function [4]. Metabolic pathways involved in lipid metabolism, amino acid utilization, and energy homeostasis have been shown to influence inflammatory responses in several immune-mediated diseases [5]. In AS, however, the extent to which metabolic alterations interact with immune dysregulation has not been comprehensively explored.

Recent advances in transcriptomic technologies and data integration methods have enabled systematic examination of disease-associated molecular changes [6]. By integrating multiple independent datasets, we can improve the consistency of detected signals and reduce bias from individual datasets. Combined with curated gene annotations for metabolism and immune functions, this approach helps identify coordinated biological modules relevant to ankylosing spondylitis [7]. Machine learning has become a useful tool for analyzing high-dimensional biological data, particularly for feature selection and hub gene identification [8]. In this way, we can prioritize genes that appear to play important roles in disease-related molecular networks [9].

In this study, we integrated multiple transcriptomic datasets to investigate metabolism-related molecular features in ankylosing spondylitis. Weighted gene co-expression network analysis (WGCNA) was employed to identify metabolism-associated genes, which were further refined through eleven machine learning algorithms to construct diagnostic models and select hub genes. The biological relevance of these genes was further examined through immune cell infiltration analysis, metabolic pathway activity assessment, and validation using single-cell transcriptomic data. In addition, post-transcriptional regulatory relationships were explored by constructing a competing endogenous RNA network. Finally, in silico compound prioritization was used to screen natural compounds derived from traditional Chinese medicines for potential interactions with identified hub genes. This integrative analysis offers a systems-level view of the interplay between metabolic and immune dysregulation in AS, and identifies candidate biomarkers and regulatory interactions that merit experimental follow-up.

## 2. Results

### 2.1. Identification of Differentially Expressed Genes

**Figure 1:**
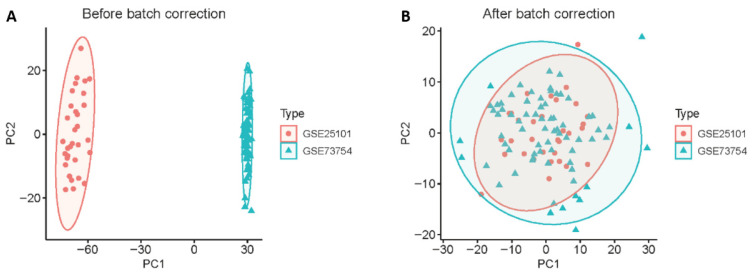
Batch effect correction and principal component analysis of merged datasets. (**A**) Principal component analysis (PCA) plot before batch correction, showing distinct clustering by dataset origin (GSE25101 in red, GSE73754 in green). (**B**) PCA plot after ComBat correction demonstrating successful removal of batch effects, with samples clustering primarily by biological variation rather than dataset origin. Each point represents one sample.

**Figure 2:**
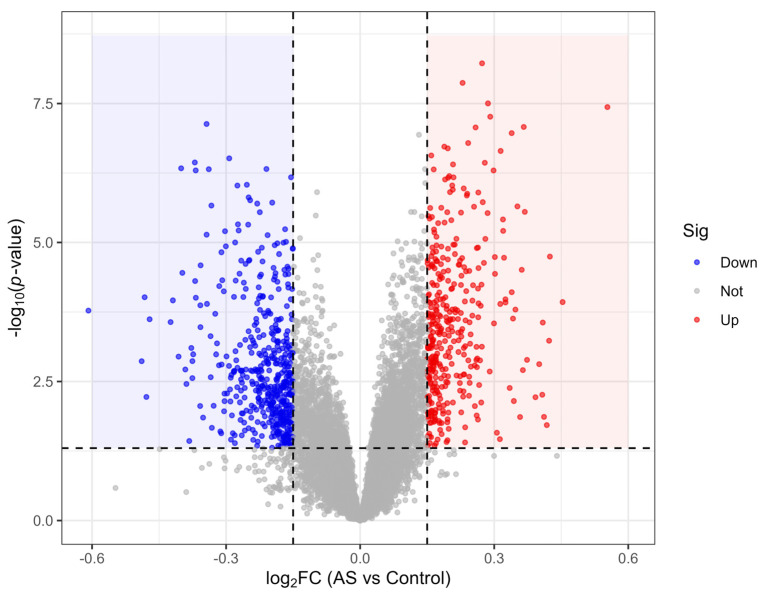
Volcano plot of differentially expressed genes between AS patients and healthy controls. Volcano plot displaying the distribution of 847 differentially expressed genes (DEGs). Red points represent upregulated genes (*n* = 364), blue points represent downregulated genes (*n* = 483). Grey points represent genes with no significant change. The horizontal dashed line indicates the significance threshold (*p* = 0.05). Vertical dashed lines are positioned at |log_2_FC| = 0.15. The blue and red shaded areas highlight the regions meeting both significance and fold-change thresholds for downregulated and upregulated genes, respectively.

Principal component analysis showed that samples clustered by disease status rather than dataset origin after batch correction (Figure 1). Differential expression analysis identified 847 genes between AS patients and healthy controls, including 364 upregulated and 483 downregulated genes (*p* < 0.05, |log_2_FC| > 0.15) (Figure 2). The expression patterns of all DEGs are shown in Figure S1.

### 2.2. WGCNA Module Identification and Metabolism-Related Gene Selection

**Figure 3:**
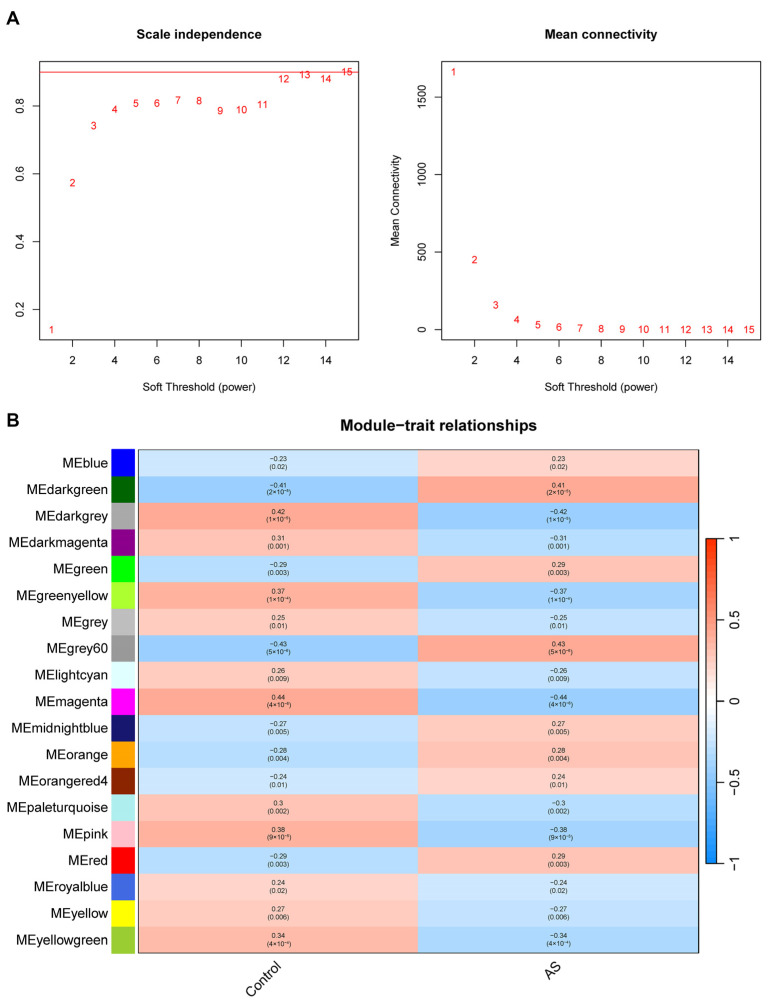
WGCNA soft-thresholding power selection and module-trait correlation analysis. (**A**) Analysis of scale-free topology fit (**left** panel) and mean connectivity (**right** panel) across different soft-thresholding powers. Red numbers indicate candidate soft-thresholding powers, and the red horizontal line marks the R^2^ = 0.9 threshold. (**B**) Heatmap showing Pearson correlation coefficients between module eigengenes (MEs) and the sample groups (AS patients and healthy controls). Each cell displays the correlation coefficient with its *p*-value in parentheses. Based on the threshold (|correlation with AS| ≥ 0.4, *p* < 0.05), the MEdarkgreen and MEgrey60 modules were considered positively correlated with AS, and the MEdarkgrey and MEmagenta modules were considered negatively correlated.

Weighted gene co-expression network analysis (WGCNA) was performed to identify co-expressed gene modules associated with the AS phenotype. A soft-thresholding power of 12 was selected to achieve scale-free topology (R^2^ = 0.9) (Figure 3A).

Module eigengenes (MEs), defined as the first principal component of each module’s gene expression matrix, were calculated to represent the overall expression profile of each module. Several modules showed significant correlations with AS. Applying a threshold of |correlation with AS| ≥ 0.4 and *p* < 0.05, we identified the MEdarkgreen (r = 0.41, 81 genes) and MEgrey60 (r = 0.43, 100 genes) modules as positively correlated with AS, while the MEdarkgrey (r = −0.42, 72 genes) and MEmagenta (r = −0.44, 205 genes) modules were negatively correlated (Figure 3B). Modules in Figure 3B are ordered by the absolute value of their correlation coefficient with AS.

**Figure 4:**
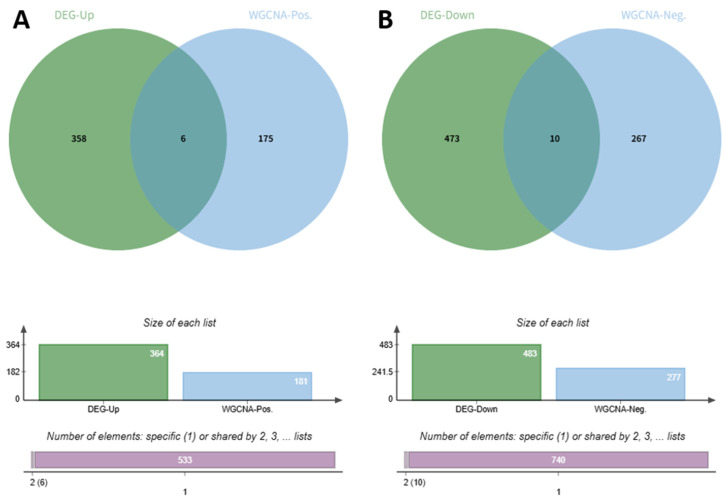
Venn diagram analysis and metabolism-related gene filtering. (**A**) Intersection of upregulated DEGs with positively correlated WGCNA modules (MEdarkgreen and MEgrey60), showing 6 overlapping genes. (**B**) Intersection of downregulated DEGs with negatively correlated WGCNA modules (MEdarkgrey and MEmagenta), showing 10 overlapping genes. The lower panel illustrates the intersection of the 16 phenotype-associated genes with metabolism-related genes annotated in the GeneCards database, confirming that all 16 candidates are metabolism-related. Bar elements indicate the size of each input gene set and the number of shared genes.

To focus on disease-relevant genes, we identified overlaps between differentially expressed genes (DEGs) and phenotype-correlated modules. Venn diagram analysis showed that 6 upregulated DEGs overlapped with genes from the positively correlated modules, and 10 downregulated DEGs overlapped with genes from the negatively correlated modules (Figure 4). All 16 of these phenotype-associated genes were confirmed as metabolism-related in the GeneCards database, forming the final candidate set for subsequent analysis.

### 2.3. Functional Enrichment Reveals RNA Metabolism and Mitochondrial Dysfunction

**Figure 5:**
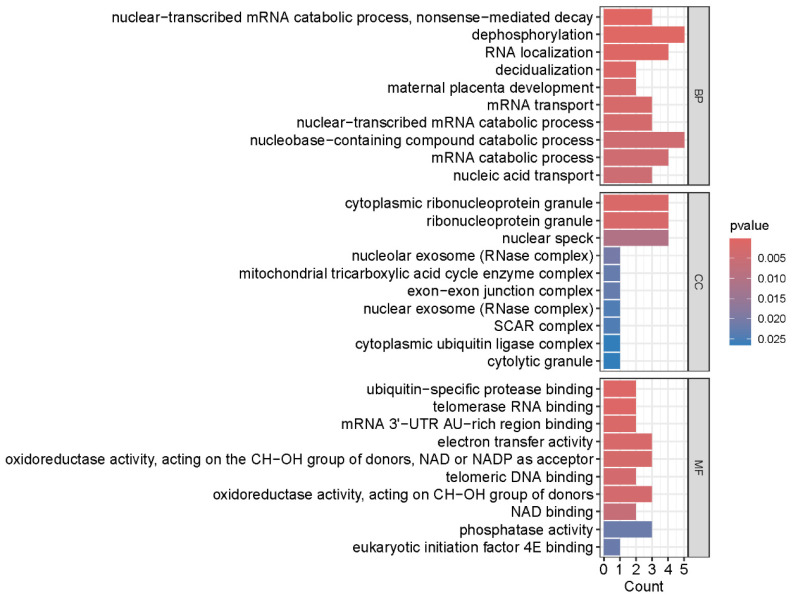
Gene Ontology enrichment analysis of metabolism-related candidate genes. Bar plot showing significantly enriched GO terms (*p* ≤ 0.025) across biological process (BP), cellular component (CC), and molecular function (MF) categories. The *x*-axis represents the gene count for each term, and the color intensity of bars corresponds to the statistical significance (−log10 (*p*-value)). Representative enriched terms include nucleic acid transport and nonsense-mediated decay (BP); cytolytic granule and mitochondrial tricarboxylic acid cycle enzyme complex (CC); and eukaryotic initiation factor 4E (eIF4E) binding, phosphatase activity, and oxidoreductase activity (MF).

Gene Ontology (GO) enrichment analysis of the metabolism-related candidate genes revealed significant enrichment (*p* ≤ 0.025) across multiple terms (Figure 5). At the biological process level, genes were predominantly enriched in pathways including nucleic acid transport, nuclear-transcribed mRNA catabolic process (nonsense-mediated decay), mRNA transport, and RNA localization. Enrichment was also observed for decidualization and maternal placenta development. The cellular component analysis showed enrichment in cytolytic granules, nuclear and nucleolar exosome complexes, ribonucleoprotein granules, and mitochondrial tricarboxylic acid cycle enzyme complexes. Molecular function categories included eukaryotic initiation factor 4E (eIF4E) binding, phosphatase activity, NAD binding, oxidoreductase activity acting on CH-OH groups with NAD/NADP as acceptors, electron transfer activity, and telomerase RNA binding.

### 2.4. Machine Learning-Based Biomarker Selection and Model Validation

**Figure 6:**
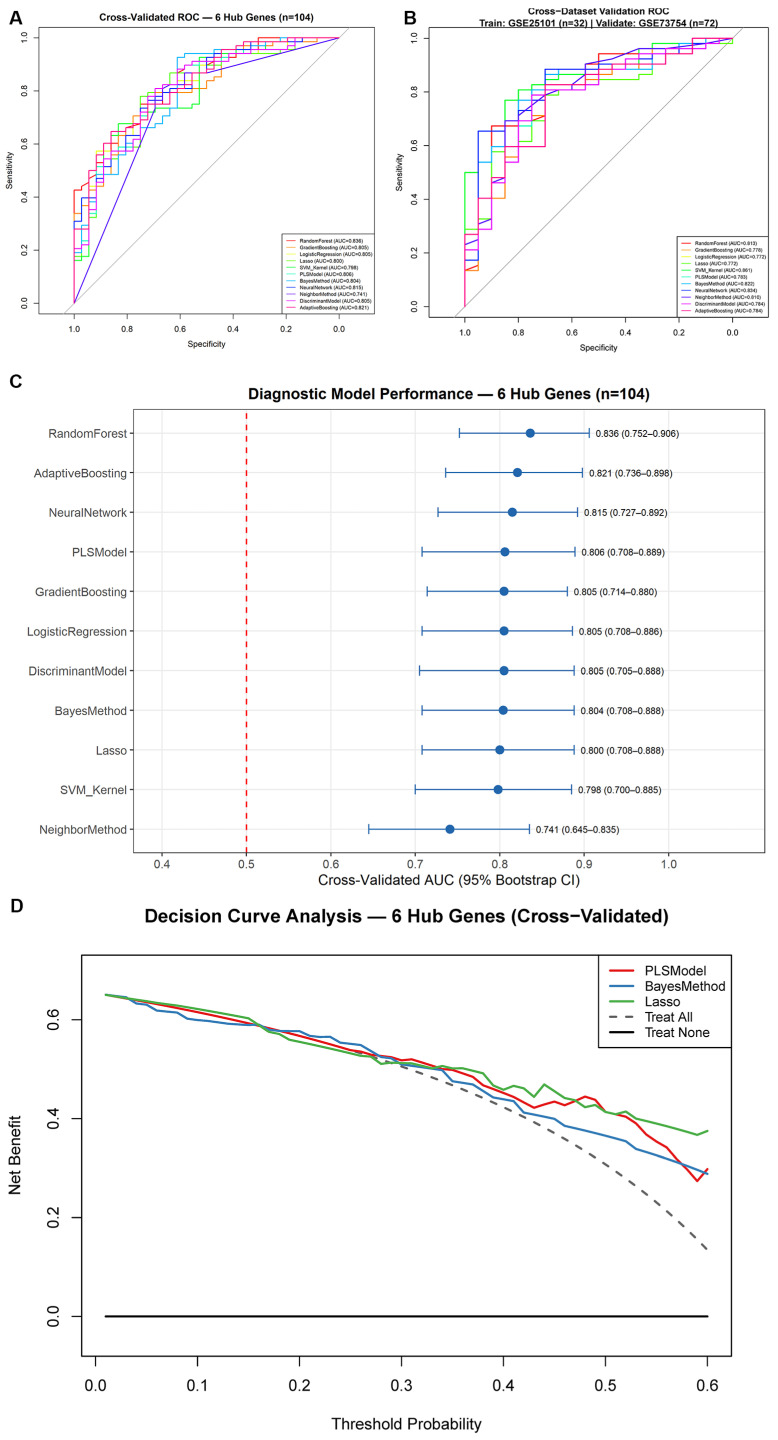
Comprehensive evaluation of 11 machine learning models for AS diagnosis. (**A**) Receiver operating characteristic (ROC) curves based on cross-validated out-of-fold predictions from the merged whole-blood cohort (*n* = 104). RandomForest achieved the highest AUC (0.836). (**B**) ROC curves from leave-one-dataset-out Round 1 validation (train: GSE25101, *n* = 32; validate: GSE73754, *n* = 72). SVM_Kernel achieved the highest validation AUC of 0.861 (95% CI: 0.767–0.938). Round 2 results (train: GSE73754; validate: GSE25101, *n* = 32) are provided in Figure S3. (**C**) Forest plot showing cross-validated AUC with 95% bootstrap confidence intervals. RandomForest achieved the highest CV AUC (0.836, 95% CI: 0.752–0.906), with overlapping confidence intervals across top-performing models indicating comparable discriminative ability. The red dashed line indicates AUC = 0.5 (random classification). (**D**) Decision curve analysis of PLSModel, BayesMethod, and Lasso based on cross-validated predictions. “Treat none” and “treat all” reference strategies are defined in the main text. Across threshold probabilities of 0.0–0.6, all three models provided net benefit above both reference strategies, suggesting informative discriminatory value within the current analytical setting. Given the exploratory nature and limited sample size, these results should not be interpreted as evidence of clinical utility.

Machine learning algorithms were trained and evaluated on the merged whole-blood cohort (*n* = 104) using 5-fold cross-validation, with cross-dataset generalizability assessed through leave-one-dataset-out (LODO) validation. All 11 algorithms were evaluated (Figure 6A; cross-validated performance metrics summarized in Figure S2). Under 5-fold cross-validation, RandomForest achieved the highest CV AUC of 0.836 (95% CI: 0.752–0.906), followed by AdaptiveBoosting (0.821, 95% CI: 0.736–0.898), NeuralNetwork (0.815, 95% CI: 0.727–0.892), PLSModel (0.806, 95% CI: 0.708–0.889), and LogisticRegression/GradientBoosting/DiscriminantModel (all 0.805) (Figure 6C and Figure S2). All top-performing models exhibited overlapping confidence intervals, suggesting comparable internal discriminative ability.

To assess cross-dataset generalizability, leave-one-dataset-out validation was performed between GSE25101 (*n* = 32) and GSE73754 (*n* = 72). In Round 1 (train: GSE25101, validate: GSE73754), SVM_Kernel achieved the highest validation AUC of 0.861 (95% CI: 0.767–0.938), followed by NeuralNetwork (0.834, 0.719–0.925) and BayesMethod (0.822, 0.703–0.919) (Figure 6B). In Round 2 (train: GSE73754, validate: GSE25101), GradientBoosting achieved the highest validation AUC of 0.715 (95% CI: 0.512–0.906). The asymmetry between rounds reflects sample size differences and inter-cohort heterogeneity. Detailed LODO validation metrics for all 11 algorithms are provided in Figure S3.

In decision curve analysis, the “treat none” strategy assumes no patient is classified as AS (net benefit = 0), while the “treat all” strategy assumes every patient is classified as AS; a model demonstrates informative value when its net benefit exceeds both references. Decision curve analysis (DCA) based on cross-validated predictions showed exploratory net-benefit patterns for PLSModel, BayesMethod, and Lasso relative to the “treat all” and “treat none” reference strategies across threshold probabilities of 0–0.6; given the exploratory nature of these analyses, these findings should be interpreted as exploratory rather than evidence of clinical utility (Figure 6D). Although RandomForest yielded a marginally higher CV AUC (0.836 vs. 0.806), the top six performing algorithms exhibited overlapping 95% confidence intervals, indicating statistically comparable discriminative ability. We selected the PLS model for downstream SHAP-based feature importance analysis because its linear additive structure allows SHAP contributions to be interpreted globally without the interaction ambiguities that arise in tree-based or deep models, thereby yielding more stable and interpretable feature rankings (Figure S4).

**Figure 7:**
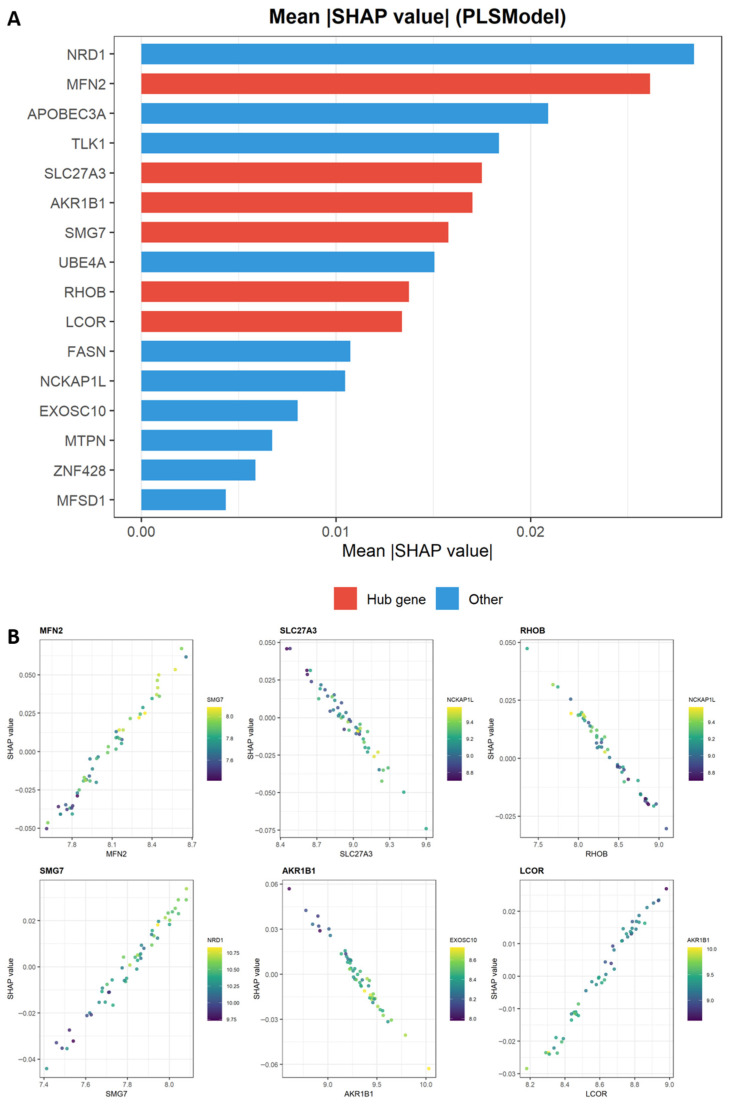
SHapley Additive exPlanations (SHAP) analysis identifies six core diagnostic biomarkers. (**A**) Bar plot of the 16 metabolism-related candidate features ranked by their mean absolute SHAP values derived from the PLS model. *NRD1* ranked highest overall (mean |SHAP| = 0.028), followed by *MFN2* (0.026). Among the six hub genes (red), *MFN2* ranked first (0.026), followed by *SLC27A3* (0.018), *AKR1B1* (0.017), *SMG7* (0.016), *RHOB* (0.014), and *LCOR* (0.013). All six hub genes were among the top 10 features. (**B**) SHAP dependence plots for the six hub genes. Each point represents one sample; the *x*-axis shows gene expression level and the *y*-axis shows the corresponding SHAP value indicating that feature’s marginal contribution to model prediction. Color indicates the expression level of the most strongly interacting feature: *MFN2-SMG7*, *SLC27A3-NCKAP1L*, *RHOB-NCKAP1L*, *SMG7-NRD1*, *AKR1B1-EXOSC10*, *LCOR-AKR1B1*. Note that SHAP dependence directions reflect multivariate model contributions and may differ from univariate differential expression directions (log_2_FC), as the model captures complex inter-gene interaction patterns rather than individual gene-level mean differences.

SHAP analysis identified *MFN2* as the top-ranking feature (mean |SHAP| = 0.026) among the six hub genes, followed by *SLC27A3* (0.018), *AKR1B1* (0.017), *SMG7* (0.016), *RHOB* (0.014), and *LCOR* (0.013). All six hub genes were confirmed among the top 10 features out of 16 candidates (Figure 7A). SHAP dependence plots revealed distinct expression-contribution relationships for each hub gene within the multivariate diagnostic model. Notably, SHAP-derived feature contribution directions reflect the joint discriminative patterns learned by the model and may not correspond directly to univariate differential expression directions, as the model integrates complex inter-gene interactions. (Figure 7B and Figure S5, Supplementary Table S1).

### 2.5. Immune Landscape Alterations and Gene-Immune Associations in AS

Given the autoimmune etiology of AS, we employed CIBERSORT deconvolution to estimate the relative abundance of 22 immune cell types from bulk transcriptomic data. Comparative analysis revealed significant alterations in the peripheral immune landscape of AS patients compared to healthy controls (Figure S6). Most notably, AS patients showed a marked decrease in the proportion of CD8+ T cells (*p* < 0.01). Concurrently, an increased frequency of CD4+ memory activated T cells (*p* < 0.05) and a reciprocal decrease in CD4+ memory resting T cells (*p* < 0.05) were observed. Additionally, a non-significant increase in neutrophil abundance and alterations in macrophage polarization states were noted, though with considerable inter-individual variability.

**Figure 8:**
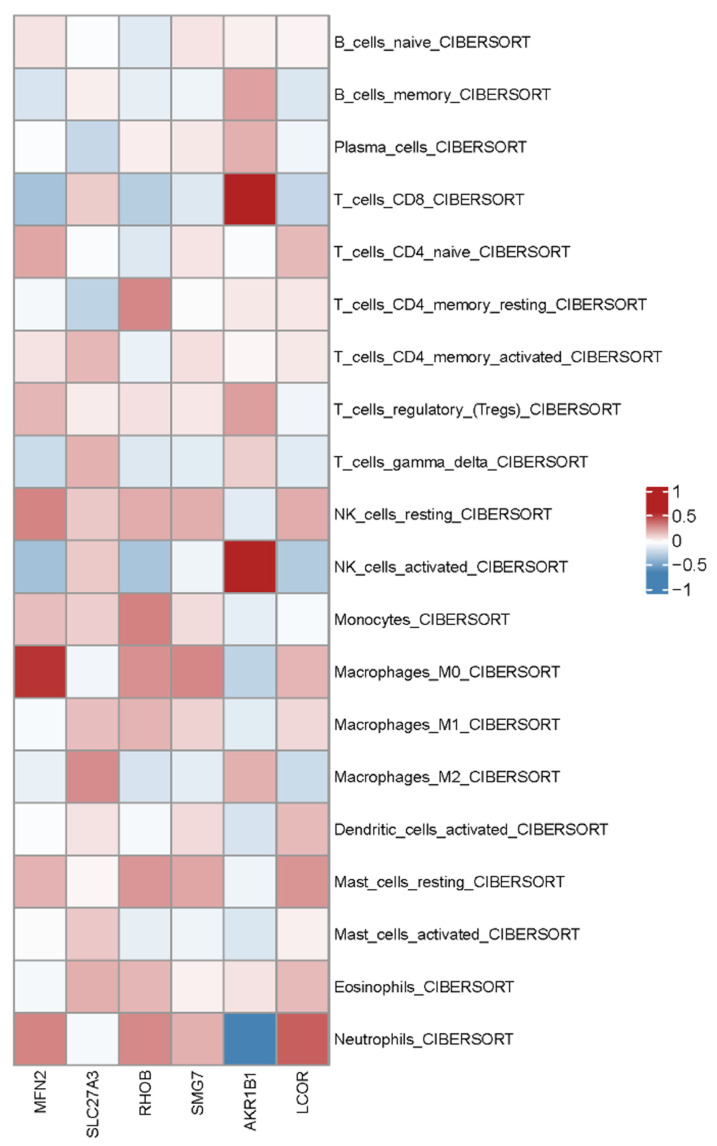
Spearman correlation between hub gene expression and immune cell infiltration. Heatmap of Spearman correlation coefficients between the expression levels of the six hub genes (*MFN2*, *SLC27A3*, *RHOB*, *SMG7*, *AKR1B1*, *LCOR*) and the infiltration scores of 22 immune cell types estimated by CIBERSORT. Notable correlations include *MFN2*–CD8+ T cells (positive), *SLC27A3*–neutrophils (positive), and *RHOB*–regulatory T cells (negative). Group-level comparisons of immune cell proportions are provided in Figure S6.

To explore functional links between hub genes and immune dysregulation, we performed Spearman correlation analysis between the expression of the six hub genes and the infiltration scores of differentially abundant immune cells (Figure 8). *MFN2* expression showed a strong positive correlation with CD8+ T cell abundance (r > 0.5, *p* < 0.01) and a negative correlation with M2 macrophages (r < −0.4, *p* < 0.05). *SLC27A3* expression correlated positively with neutrophil abundance (r > 0.6, *p* < 0.001). *RHOB* expression was positively correlated with M1 macrophage abundance (r > 0.4, *p* < 0.05) and negatively correlated with regulatory T cells (Tregs) (r < −0.5, *p* < 0.01). The remaining hub genes (*SMG7*, *AKR1B1*, *LCOR*) also exhibited statistically significant correlations with specific immune subsets.

### 2.6. Metabolic Pathway Dysregulation and Hub Gene-Pathway Correlations in AS

To characterize metabolic reprogramming, we applied single-sample gene set enrichment analysis (ssGSEA) to quantify the activity of 86 metabolic pathways. This revealed significant alterations in AS patients compared to controls (Figure S7). Three core energy metabolism pathways—oxidative phosphorylation, the tricarboxylic acid (TCA) cycle, and glycolysis—were simultaneously downregulated (*p* < 0.01). Concurrently, oxidative stress-related pathways, including glyoxylate and glutathione metabolism, were markedly upregulated (*p* < 0.001). Alterations were also observed in amino acid, fatty acid, and nucleotide metabolism pathways.

**Figure 9:**
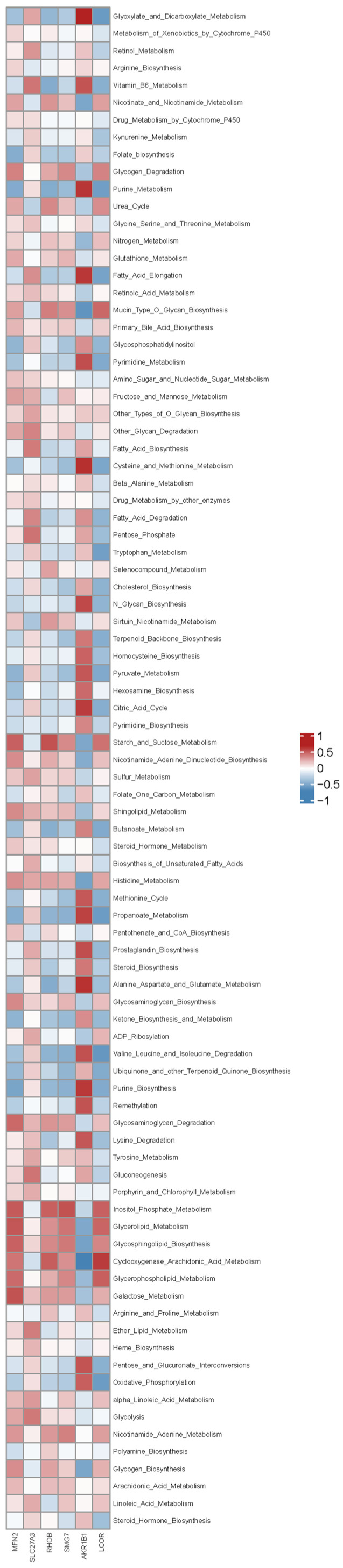
Spearman correlation between hub gene expression and metabolic pathway activity. Heatmap of Spearman correlation coefficients between hub gene expression and metabolic pathway scores quantified by ssGSEA. Notable correlations include *MFN2* with oxidative phosphorylation (r > 0.6) and the TCA cycle; *SLC27A3* with fatty acid degradation and ketone biosynthesis; and *AKR1B1* with the pentose phosphate pathway. Group-level comparisons of pathway activity are provided in Figure S7.

To determine associations between hub gene expression and these pathway-level alterations, we performed Spearman correlation analysis between the six hub genes and the metabolic pathway scores (Figure 9). *MFN2* expression showed strong positive correlations with oxidative phosphorylation (r > 0.6, *p* < 0.001) and TCA cycle activity (r > 0.5, *p* < 0.001). *SLC27A3* correlated positively with fatty acid degradation (r > 0.7, *p* < 0.001) and ketone biosynthesis (r > 0.5, *p* < 0.01). *AKR1B1* expression was strongly correlated with pentose phosphate pathway activity (r > 0.7, *p* < 0.001). *SMG7* and *LCOR* exhibited negative correlations with multiple anabolic pathways, while *RHOB* showed correlations with diverse metabolic processes (all *p* < 0.05).

### 2.7. Supportive Single-Cell Analysis Reveals Cell-Type-Associated Hub Gene Expression Patterns in AS

**Figure 10:**
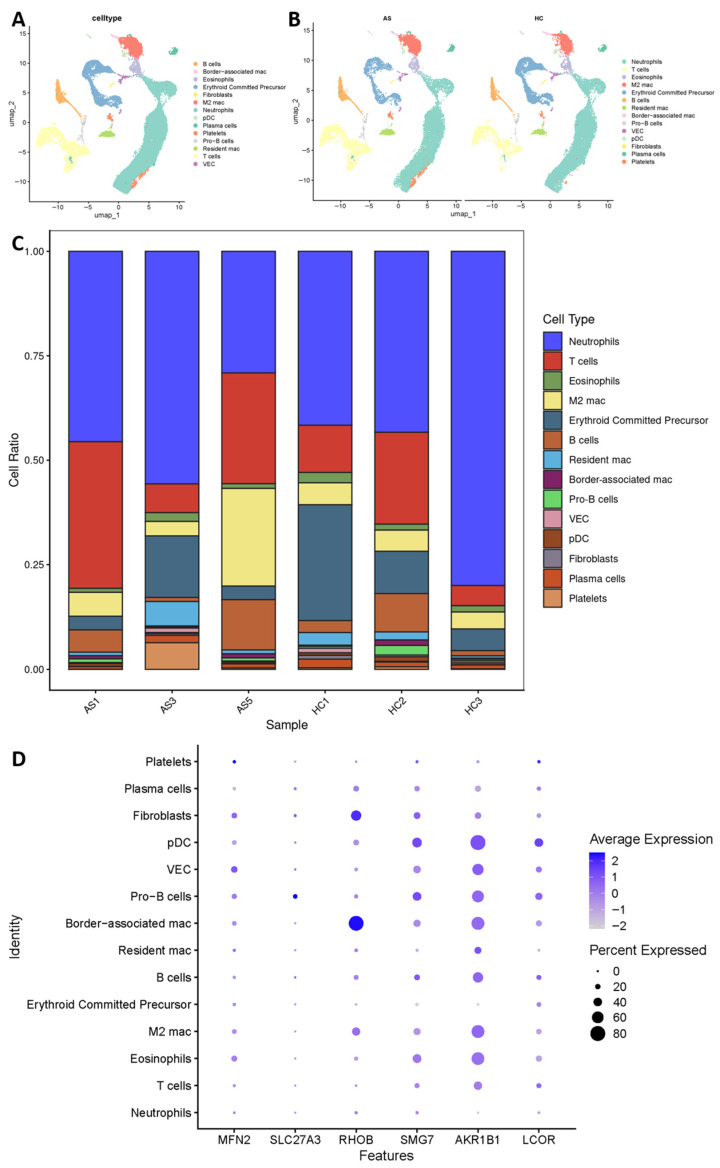
Supportive single cell RNA-seq analysis of peripheral blood from AS patients and healthy controls. (**A**) UMAP plot displaying 14 distinct cell populations identified by unsupervised clustering (*n* = 6 samples: 3 AS, 3 HC). Cell types include neutrophils, T cells, eosinophils, M2 macrophages, erythroid committed precursor cells, B cells, resident macrophages, border-associated macrophages, pro-B cells, vascular endothelial cells (VEC), plasmacytoid dendritic cells (pDC), fibroblasts, plasma cells, and platelets. (**B**) UMAP plots split by disease status (AS vs. HC) showing compositional differences. (**C**) Stacked bar plot quantifying cell type proportions across individual samples. Neutrophils constitute approximately 55% of cells in AS patients versus 35% in controls, accompanied by a corresponding decrease in T cells. (**D**) Dot plot illustrating hub gene expression patterns across the 14 cell types. *RHOB* shows highest expression in border-associated macrophages and fibroblasts. *AKR1B1* is broadly expressed with highest abundance in pDCs, VECs, and pro-B cells.

To provide supportive single-cell-level evidence, we analyzed scRNA-seq data from peripheral blood of 3 AS patients and 3 healthy controls (GSE268839). After quality control and batch correction, unsupervised clustering identified 14 distinct cell populations (Figure 10A and Figure S8).

Comparative analysis suggested apparent shifts in cell type proportions (Figure 10B,C). Neutrophils constituted approximately 55% of total cells in AS patients versus 35% in controls. This expansion was accompanied by a reciprocal reduction in T cell proportions.

Hub gene expression exhibited cell type-specific patterns (Figure 10D). *RHOB* expression was highest in border-associated macrophages and fibroblasts. *AKR1B1* showed broad expression, with highest abundance in plasmacytoid dendritic cells (pDCs), vascular endothelial cells (VECs), and pro-B cells. *MFN2* and *SMG7* expression was more restricted to lymphoid lineages (e.g., T cells and B cells), while *SLC27A3* and *LCOR* showed enrichment in specific myeloid subsets (e.g., neutrophils).

Single-cell ssGSEA analysis revealed cell type-specific metabolic activities (Figure S9). T cells displayed high oxidative phosphorylation scores. In contrast, neutrophils showed high glycolytic scores. In this exploratory dataset, AS-derived neutrophils exhibited significantly higher glycolytic pathway scores than control neutrophils.

### 2.8. Hub Gene Functional Characterization Through Pathway Enrichment Analysis

To elucidate the biological functions associated with each hub gene, we applied two complementary approaches: Gene Set Enrichment Analysis (GSEA) on samples stratified into high- and low-expression groups for each hub gene, and Gene Set Variation Analysis (GSVA) to compute sample-level pathway activity scores that were then correlated with hub gene expression.

*MFN2* high-expression samples were positively enriched for mitochondrial gene expression, mitochondrial translation, and oxidative phosphorylation (Figure S10), consistent with *MFN2*’s canonical role in mitochondrial fusion and bioenergetics. At the sample level, *MFN2* expression correlated positively with leukocyte transendothelial migration, Fc gamma R–mediated phagocytosis, focal adhesion, and regulation of the actin cytoskeleton, and negatively with pyruvate metabolism, ribosome, and protein export (Figure S11).

*SMG7* and *LCOR* high-expression samples were negatively enriched in oxidative phosphorylation, ribosome biogenesis, and mitochondrial translation (Figures S12 and S13), with *SMG7* additionally linked to downregulation of ribosomal and spliceosome pathways. *LCOR* upregulation was positively associated with JAK-STAT and MAPK signaling (Figures S13 and S14), whereas *SMG7* activity correlated positively with mTOR signaling and focal adhesion and negatively with proteasome and T-cell receptor signaling (Figure S15).

*SLC27A3* high-expression samples were positively enriched in allograft rejection, antigen processing and presentation, asthma, leishmania infection, and type I diabetes mellitus, while ribosome-related terms were negatively enriched (Figure S16); consistent with an antiviral/immunoregulatory profile, *SLC27A3* expression also correlated positively with the cytosolic DNA-sensing and RIG-I-like receptor signaling pathways and sulfur metabolism, and negatively with Wnt and phosphatidylinositol signaling (Figure S17). *RHOB* showed a coherent pattern across both analyses, with positive associations to chemokine signaling, leukocyte transendothelial migration, and regulation of the actin cytoskeleton (Figures S18 and S19). For *AKR1B1*, the two analyses converged on ribosome- and rRNA-related terms—positive enrichment in ribosome and DNA replication (Figure S20) alongside positive correlations with ribosome biogenesis, rRNA metabolism, and aminoacyl-tRNA biosynthesis (Figure S21)—but diverged on immune pathways: Toll-like receptor signaling and complement cascades were positively enriched in the GSEA ranking (Figure S20), whereas Toll-like receptor 2 signaling, chemokine signaling, and leukocyte transendothelial migration were negatively correlated in the sample-level GSVA (Figure S21). This divergence reflects the methodological difference between rank-based enrichment and sample-level pathway scoring rather than contradictory biological evidence.

### 2.9. Chromosomal Co-Localization of Hub Genes

**Figure 11:**
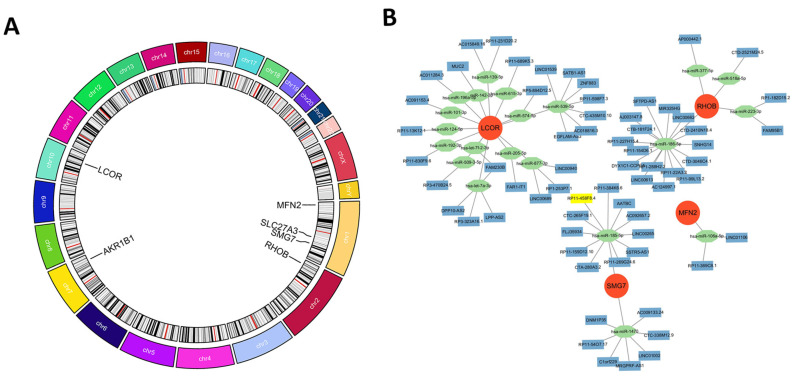
Genomic distribution and post-transcriptional regulation of hub genes. (**A**) Circos plot illustrating the chromosomal positions of the six hub genes. Three genes (*MFN2*, *SLC27A3*, *SMG7*) are located on chromosome 1. The remaining genes are positioned on chromosome 2 (*RHOB*), chromosome 7 (*AKR1B1*), and chromosome 10 (*LCOR*). (**B**) Competing endogenous RNA (ceRNA) regulatory network. Orange nodes represent hub gene mRNAs, green nodes represent miRNAs, and light blue nodes represent lncRNAs. The network comprises 6 hub gene mRNAs, 21 miRNAs, and 65 lncRNAs, connected by 86 edges. Key predicted regulatory axes include *SATB1-AS1*/miR-539-5p/*LCOR*, *FAM95B1*/miR-223-3p/*RHOB*, *LINC01106*/miR-106a-5p/*MFN2*, and *AATBC*/miR-185-5p/*SMG7*. Within this network, *LCOR* interacts with 12 miRNAs, and miR-186-5p connects 30 lncRNAs and targets both *RHOB* and *SMG7*. Abbreviations: chr, chromosome; miRNA, microRNA; lncRNA, long non-coding RNA; mRNA, messenger RNA.

Genomic mapping revealed a non-random distribution pattern of the six hub genes (Figure 11A). Specifically, three genes—*MFN2* (1p36.22), *SLC27A3* (1q21.3), and *SMG7* (1q25.2)—are located on chromosome 1, indicating spatial clustering within the same chromosome.

The remaining genes are distributed across other autosomes: *RHOB* on chromosome 2 (2p24.1), *AKR1B1* on chromosome 7 (7q35), and *LCOR* on chromosome 10 (10q24.31).

Further analysis of genomic context showed that *AKR1B1* resides in a tandem array with its paralog *AKR1B10* on 7q35. On chromosome 10, the *LCOR* locus (10q24.31) is positioned approximately 1.6 megabases distal to the *PTEN* gene locus (10q23.31).

### 2.10. Competitive Endogenous RNA Network Reveals Multi-Layered Post-Transcriptional Control

To investigate post-transcriptional regulatory mechanisms, we constructed a competitive endogenous RNA (ceRNA) network. Through stringent database filtering, we identified 21 miRNAs targeting the six hub genes and 65 lncRNAs predicted to sequester these miRNAs, forming a network of 92 nodes and 86 edges (Figure 11B).

Network topology analysis revealed that *LCOR* is targeted by 12 distinct miRNAs, including miR-539-5p, miR-205-5p, miR-139-5p, and let-7 family members. Among miRNAs, miR-186-5p connects to 30 lncRNAs and targets both *RHOB* and *SMG7*. The analysis identified four prominent regulatory axes: *SATB1-AS1*/miR-539-5p/*LCOR*, *FAM95B1*/miR-223-3p/*RHOB*, *LINC01106*/miR-106a-5p/*MFN2*, and *AATBC*/miR-185-5p/*SMG7*.

### 2.11. Identification of Natural Compounds Targeting Hub Genes

To identify potential therapeutic compounds, we compiled 66 traditional Chinese medicines commonly used for AS treatment based on the literature. Chemical components were retrieved from TCMSP and BATMAN-TCM databases, yielding 4012 compounds. Finally, 2685 compounds with available structural information in PubChem were selected for binding analysis. Deep learning-based prediction was used to calculate binding scores between these compounds and the six hub genes.

**Figure 12:**
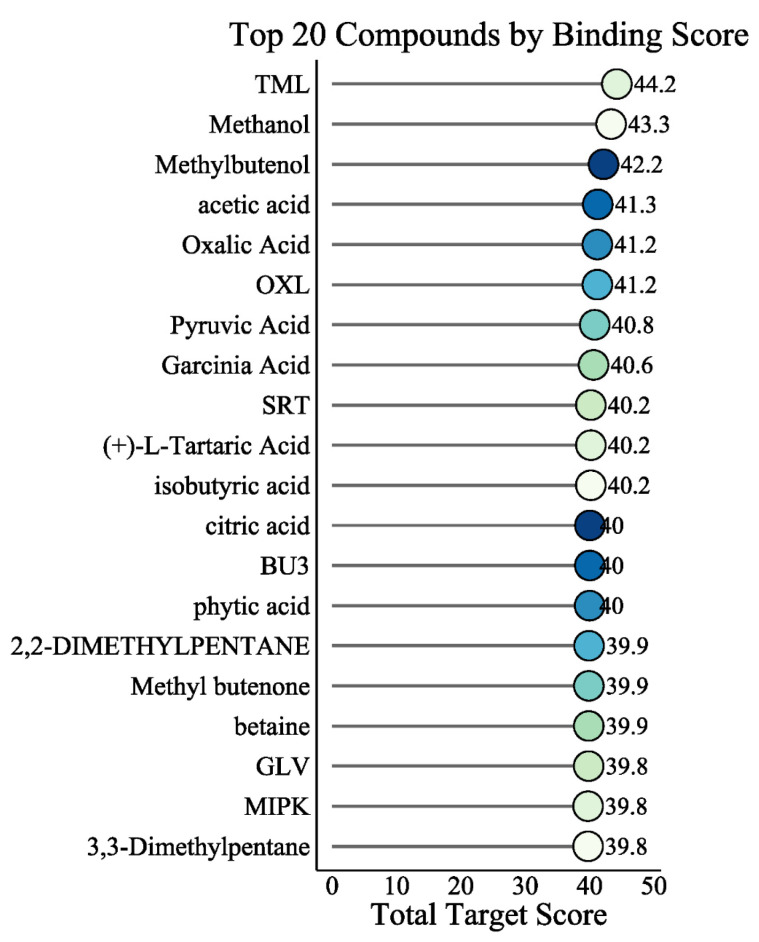
In silico prioritization of candidate compounds targeting hub-gene-encoded proteins. Bar chart of the top 20 predicted compounds ranked by total binding score. High-scoring bioactive candidates including betaine, pyruvic acid, and citric acid are indicated. These compounds were prioritized based on model-predicted interaction scores and should not be interpreted as validated binders or therapeutic agents. Dot colors are used for visual distinction.

The analysis revealed that the protein encoded by *MFN2* exhibited the highest overall binding capacity with the compound library. Among the top-ranking compounds, several known bioactive molecules with favorable safety profiles were identified. Betaine achieved a high total binding score (39.9), alongside other metabolites with established biological activities, including pyruvic acid (40.8), citric acid (40.0), and (+)-L-tartaric acid (40.2). The complete ranked list of the top 20 compounds is provided in Figure 12. The complete ranked output of the compound prioritization analysis is provided in Supplementary Table S2, including PubChem CIDs, source TCM annotations, total prioritization scores, and nominal top-associated hub-gene targets.

## 3. Discussion

This study used an integrative multi-omics approach to examine the transcriptomic landscape of ankylosing spondylitis (AS), with emphasis on the relationship between metabolic pathway alterations and immune cell composition. The prevailing pathophysiological framework for AS centres on aberrant immune responses linked to HLA-B27 and the IL-23/IL-17 inflammatory axis [10,11]. However, accumulating evidence has begun to reframe AS as a condition with a significant immuno-metabolic dimension [12,13]. Our integrated bulk and single-cell transcriptomic analysis extends this perspective by identifying a six-gene candidate diagnostic signature and a set of regulatory relationships that together point to coordinated suppression of core energy-metabolism pathways as a prominent transcriptomic feature accompanying the immune dysregulation observed in AS.

A principal outcome of this analysis was the reduction in high-dimensional transcriptomic data to a compact candidate panel suitable for exploratory diagnostic evaluation. By employing Weighted Gene Co-expression Network Analysis (WGCNA) to isolate disease-correlated gene modules, followed by a rigorous 11-algorithm machine learning pipeline, we identified a concise six-gene signature (*MFN2*, *SLC27A3*, *SMG7*, *RHOB*, *AKR1B1*, *LCOR*). Among the 11 algorithms evaluated using the locked six-gene signature, RandomForest achieved the highest cross-validated AUC of 0.836 (95% CI: 0.752–0.906), followed by AdaptiveBoosting (0.821, 95% CI: 0.736–0.898) and NeuralNetwork (0.815, 95% CI: 0.727–0.892). All models showed comparable internal discriminative ability with overlapping confidence intervals. The PLS model (CV AUC = 0.806, 95% CI: 0.708–0.889) was selected for downstream SHAP analysis. To assess cross-dataset generalizability using tissue-matched data, leave-one-dataset-out (LODO) validation was performed between the two constituent whole-blood cohorts. In Round 1 (train: GSE25101, *n* = 32; validate: GSE73754, *n* = 72), SVM_Kernel achieved the highest validation AUC of 0.861 (95% CI: 0.767–0.938), with all models exceeding 0.77. In Round 2 (train: GSE73754, *n* = 72; validate: GSE25101, *n* = 32), GradientBoosting achieved the highest validation AUC of 0.715 (95% CI: 0.512–0.906), though confidence intervals were wider due to the smaller validation sample. The asymmetry between rounds reflects inter-cohort heterogeneity and sample size differences. Overall, the LODO analysis demonstrated moderate cross-cohort reproducibility of the six-gene diagnostic signature within whole-blood transcriptomic data. Based on cross-validated predictions, the DCA results indicate that the models showed exploratory net-benefit patterns over relevant threshold ranges in the current analytical setting; however, these findings should not yet be regarded as definitive evidence of clinical utility.

Our results indicate that widespread alterations in core energy-metabolism pathways constitute a prominent transcriptomic feature of AS in peripheral blood. While previous studies have linked AS risk to specific serum metabolites or focused on metabolic shifts in particular cell subsets, such as enhanced glycolysis in activated Th17 cells in other autoimmune diseases [13,14], our findings reveal a more pervasive and coordinated functional decline. We observed a simultaneous suppression of three cornerstone energy pathways: oxidative phosphorylation, the tricarboxylic acid (TCA) cycle, and glycolysis (all *p* < 0.01). This suggests that immune cells in AS may first encounter a systemic failure in energy generation, which may be associated with the diverse cell-specific metabolic and immune alterations observed in the current datasets.

The hub genes identified here may mark transcriptomic states associated with these pathway alterations. Notably, *MFN2*, a master regulator of mitochondrial dynamics, emerged as the highest-ranking hub gene by SHAP analysis (mean |SHAP| = 0.026), ranking second overall among all 16 candidate features behind only NRD1 (0.028). Its expression showed a strong positive correlation with both oxidative phosphorylation (r > 0.6, *p* < 0.001) and TCA cycle activity (r > 0.5, *p* < 0.001). Furthermore, GSEA indicated that higher *MFN2* expression was associated with mitochondrial gene expression and translation pathways, consistent with a role in the mitochondrial alterations observed in AS. In addition, our analysis identified a post-transcriptional dimension that has received limited attention in AS research. The enrichment of pathways related to nonsense-mediated mRNA decay, together with the significant correlation between *SMG7* expression and oxidative phosphorylation pathway activity, raises the possibility that altered mRNA surveillance may contribute to the metabolic suppression detected in the present datasets.

These metabolic alterations may be relevant to the immune cell changes identified in our analysis. The most notable findings were a reduced proportion of peripheral CD8+ T cells (*p* < 0.01) and an increased proportion of neutrophils, observed both in the bulk deconvolution results and, to a similar degree, in the limited single-cell dataset. The reduction in CD8+ T cells, a population known to depend on efficient oxidative phosphorylation, is consistent with the observed downregulation of *MFN2* in AS, given the positive correlation between *MFN2* expression and CD8+ T cell abundance (r > 0.5, *p* < 0.01). Conversely, the expanded neutrophil population displays a distinct metabolic signature. Our single-cell ssGSEA revealed that AS neutrophils maintain significantly higher glycolytic pathway scores, adopting a “Warburg-like” hyper-glycolytic state. This metabolic reprogramming likely provides the energy requisite for their pro-inflammatory functions and survival, thereby fueling chronic inflammation. However, this creates a critical paradox: how do specific cell expansions and high-glycolytic states coexist with the metabolic collapse observed in the periphery? Recent evidence offers a resolution. It has been proposed that under chronic antigenic stimulation, specific CD8+ T cell subsets can resist classical exhaustion by maintaining mitochondrial fitness [15,16], while dysregulation of global regulators like the FOXO1-SIRT1 axis contributes to systemic immune-metabolic coupling [4]. Building on these insights, we suggest that the initial systemic metabolic hypofunction creates a baseline environmental stress. Consequently, while the majority of OXPHOS-dependent CD8+ T cells succumb to functional exhaustion, IL-15-mediated metabolic support has been proposed as one mechanism by which a subset of CD8+ T cells may partially resist this pressure [17,18]. Simultaneously, myeloid cells such as neutrophils appear prone to shifting directly toward high-efficiency glycolysis. This divergent adaptation, likely coordinated by axes such as FOXO1-SIRT1 [4], perpetuates the inflammatory loop and offers a novel model for AS chronicity.

The dysregulation of the six hub genes appears to be entrenched by mechanisms at both the genomic and post-transcriptional levels. We observed a physical co-localization of the core hub genes *MFN2*, *SLC27A3*, and *SMG7* on chromosome 1. From a genomic perspective, this suggests they may reside within shared chromatin topological domains, coordinated by common cis-regulatory elements, providing a structural basis for their transcriptional synergy. This finding aligns with emerging functional genomics paradigms, which posit that non-coding variants influence disease susceptibility through cell-type-specific regulatory networks [19]. Our results extend this logic to the immunometabolic phenotype of AS: physically clustered genes likely undergo coordinated dysregulation, thereby amplifying downstream systemic metabolic failure. Crucially, our ceRNA network analysis reveals that these genes also constitute a functional module at the post-transcriptional level. We found that co-located hub genes and other key nodes are targeted by shared miRNAs; for instance, miR-186-5p acts as a bridge, simultaneously regulating *RHOB* and *SMG7*. The genomic co-localization and ceRNA regulation of this metabolic module ensure stability under normal conditions but represent a potentially vulnerable regulatory module in AS, disrupting this central node can trigger a systemic failure of the entire network.

Current biologic therapies for AS primarily target inflammatory cytokines such as TNF-α or IL-17, yet they do not directly address the fundamental metabolic disturbances identified in our study [20]. This therapeutic gap highlights a potential avenue for complementary or alternative therapeutic strategies. Our deep learning-based screening of 2685 natural compounds against the proteins encoded by the hub genes identified several compelling candidates. Notably, betaine, pyruvic acid, and citric acid yielded high predicted binding scores against the hub gene-encoded proteins. However, these predictions are based exclusively on in silico models, and high binding scores do not directly imply pharmacological activity. These results should be regarded solely as hypothesis-generating leads requiring experimental validation. The significance of betaine is underscored by its established mitochondrial protective function, which our analysis suggests could counteract the *MFN2*-related bioenergetic deficit. This is corroborated by previous findings that betaine promotes cellular survival via *MFN2* regulation [21]. Concurrently, its function as an osmolyte could counteract the polyol pathway stress implicated by *AKR1B1*, positioning it as a multifaceted candidate aligned with our model [22]. Furthermore, recent research has repositioned betaine beyond a simple metabolite, identifying it as an endogenous ‘exercise mimetic’ capable of systemically improving metabolic health and counteracting chronic inflammation. This effect is mediated, in part, through a defined mechanism involving direct targeting of the immune kinase TBK1 to exert anti-inflammatory effects [23]. These findings identify betaine and related endogenous metabolites as candidate compounds warranting further investigation in AS. However, we emphasize that all compound screening results were derived from computational DTI prediction using the DeepPurpose deep learning framework, and the predicted binding affinities have not been experimentally confirmed. The translational potential of these candidates must be evaluated through in vitro binding assays (e.g., surface plasmon resonance, isothermal titration calorimetry), cellular functional studies, and preclinical models before any therapeutic relevance can be established. If validated, such metabolite-based approaches aimed at restoring the metabolic–immune interface could potentially complement existing anti-inflammatory treatments.

While this integrative analysis suggests a potential immunometabolic framework for AS, we acknowledge its limitations. Our findings are primarily derived from transcriptomic data. Future studies employing proteomic and metabolomic profiling are essential to confirm whether these observed changes in gene expression translate to corresponding alterations at the protein and functional metabolite levels [24]. The predicted ceRNA network interactions and the compound-target binding affinities are based on computational inference and require direct experimental validation, for example, through luciferase reporter assays, RNA immunoprecipitation, and in vitro binding assays. Finally, while our diagnostic signature showed robust cross-validated performance (CV AUC = 0.741–0.836), cross-dataset generalizability was assessed through leave-one-dataset-out validation between GSE25101 and GSE73754. Round 1 (validate: GSE73754, *n* = 72) yielded strong results with validation AUC ranging from 0.772 to 0.861 and all confidence interval lower bounds at or above 0.65. Round 2 (validate: GSE25101, *n* = 32) yielded more modest performance (validation AUC 0.594–0.715) with substantially wider confidence intervals. Notably, the lower 95% CI bound of the best-performing model (0.512) approaches the chance level, indicating that cross-dataset generalizability in this direction cannot yet be confidently established from the available data. The uniformly low specificity observed in Round 2 is consistent with the combined impact of class imbalance and small sample size (*n* = 32) on probability threshold calibration. Accordingly, the Round 2 validation estimate should be regarded as provisional, and the signature’s cross-cohort robustness requires reassessment before firm diagnostic claims can be made. Additionally, because batch correction (ComBat) was performed on the merged dataset prior to LODO splitting, the held-out cohort is not fully independent, and the resulting LODO metrics should be interpreted as estimates of cross-dataset reproducibility rather than strict independent validation. Exploratory application to GSE11886 (macrophage-derived samples) confirmed that the whole-blood signature does not generalize to distinct tissue types. Future work should prioritize prospective, multi-center cohort studies complemented by orthogonal validation at the protein and metabolite levels, together with functional and mechanistic investigations in primary immune cells and relevant disease models, to establish the biological relevance of the proposed metabolic–immune crosstalk in ankylosing spondylitis.

## 4. Materials and Methods

### 4.1. Data Collection and Preprocessing

All datasets used in this study (GSE25101, GSE73754, GSE11886, GSE268839) were retrieved from the NCBI Gene Expression Omnibus (GEO), a publicly accessible repository. All data are de-identified and freely available for research use. The original studies obtained appropriate ethical approvals and informed consent as described in their respective publications. As this study exclusively utilized publicly available, de-identified data, additional ethical approval was not required.

Three Gene Expression Omnibus (GEO) datasets were retrieved for this study: GSE25101 (*n* = 32), GSE73754 (*n* = 72), and GSE11886 (*n* = 18), comprising a total of 122 samples from ankylosing spondylitis (AS) patients and healthy controls. The GSE25101 and GSE73754 datasets were merged and designated as the training cohort (*n* = 104). GSE11886 (*n* = 18), comprising monocyte-derived macrophage samples, was retained for exploratory cross-tissue analysis. Batch effects between datasets were corrected using the ComBat algorithm from the sva package (version 3.48.0) in R software (version 4.3.0). Principal component analysis (PCA) was performed to verify successful batch correction.

### 4.2. Differential Expression Analysis

Differential gene expression analysis was performed using the limma package (version 3.56.0). Genes with *p* < 0.05 and |log_2_ fold change (FC)| > 0.15 were considered differentially expressed genes (DEGs). This threshold was selected to balance noise reduction with retention of biologically meaningful signals, given the characteristically modest effect sizes observed in blood-based AS transcriptomic. Downstream filtering through WGCNA and machine learning further ensured biological relevance. A sensitivity analysis of DEG identification across varying |log_2_FC| thresholds was performed to confirm the robustness of the selected cutoff (Figure S22).

### 4.3. Weighted Gene Co-Expression Network Analysis (WGCNA)

To identify disease-phenotype-related genes, WGCNA was conducted with the WGCNA package (version 1.72). Genes with low expression (mean expression < 1) were removed, and the top 10,000 most variable genes were used for network construction. A soft threshold power of 12 was chosen based on the scale-free topology criterion (R^2^ = 0.9). The minimum module size was set to 30 genes. Modules with correlation coefficients ≥ 0.4 or ≤−0.4 (*p* < 0.05) with the AS phenotype were identified as positively or negatively correlated modules, respectively.

### 4.4. Identification of Metabolism-Related Genes

Venn diagram analysis was performed using Jvenn (https://jvenn.toulouse.inrae.fr/app/index.html; accessed on 30 November 2025) to identify the intersection between DEGs and WGCNA modules. Specifically, upregulated DEGs were intersected with positively correlated modules, while downregulated DEGs were intersected with negatively correlated modules. The resulting 16 phenotype-associated genes were further filtered against metabolism-related genes retrieved from the GeneCards database (https://www.genecards.org/) (search term: “Metabolism”; complete gene list in Supplementary Table S1). All 16 genes were confirmed as metabolism-related, forming the final candidate set for subsequent analyses.

### 4.5. Gene Ontology Enrichment Analysis

Gene Ontology (GO) functional enrichment analysis was performed using the clusterProfiler package (version 4.8.0) with the enrichGO function. The org.Hs.eg.db package (version 3.17.0) was used for gene annotation. Significantly enriched GO terms were defined as those with a *p*-value < 0.05. Results encompassing biological process (BP), cellular component (CC), and molecular function (MF) ontologies were visualized using the ggplot2 (version 3.4.0), enrichplot (version 1.20.0), and circlize (version 0.4.15) packages. GO functional enrichment analysis was performed on the metabolism-related candidate genes to characterize the functional landscape of the disease-associated gene set.

### 4.6. Machine Learning-Based Diagnostic Model Construction

An ensemble machine learning framework comprising 11 algorithms was implemented to identify core diagnostic biomarkers: Random Forest, Gradient Boosting, Support Vector Machine with Kernel (SVM_Kernel), Logistic Regression, K-Nearest Neighbors (NeighborMethod), Partial Least Squares (PLSModel), AdaBoost (AdaptiveBoosting), Neural Network, Naive Bayes (BayesMethod), Linear Discriminant Analysis (DiscriminantModel), and Least Absolute Shrinkage and Selection Operator (Lasso). Data centering and scaling were integrated into the model training procedure via the preProcess parameter in caret to prevent information leakage during cross-validation. Class predictions were derived from model probability estimates using a 0.5 threshold to ensure consistency across algorithms, as default class assignments from certain implementations (e.g., ada) may not align with calibrated probability outputs.

Model development proceeded in two stages. First, the 16 metabolism-related candidate genes were used for initial algorithm comparison and SHAP-based feature importance ranking, from which six hub genes were identified. Final model evaluation was then performed using the locked six-gene signature.

Model performance was assessed primarily by 5-fold cross-validated AUC with 95% confidence intervals, supplemented by sensitivity, specificity, accuracy, and F1-score. To evaluate cross-dataset generalizability using tissue-matched data, a leave-one-dataset-out (LODO) validation strategy was employed: GSE25101 and GSE73754 were alternately used as training and validation sets, with bootstrap-based 95% confidence intervals calculated for validation AUC. As an exploratory cross-tissue analysis, the six-gene signature was also applied to GSE11886; however, as this dataset comprises monocyte-derived macrophages rather than whole blood, these results are not interpreted as formal external validation. Decision curve analysis was performed on cross-validated predictions to examine net-benefit patterns across a range of threshold probabilities.

### 4.7. Immune Cell Infiltration Analysis

The relative abundance of 22 immune cell types in bulk RNA-seq data was estimated using the CIBERSORT algorithm with the LM22 signature matrix. CIBERSORT employs support vector regression (ν-SVR) to deconvolve gene expression profiles into cell type-specific proportions. The algorithm was executed with 1000 permutations for significance testing, and only samples with a CIBERSORT *p*-value < 0.05 were retained to ensure high-confidence deconvolution results.

Group-wise comparisons of immune cell infiltration between AS patients and healthy controls were performed using the Wilcoxon rank-sum test, with statistical significance set at α = 0.05 (* *p* < 0.05, ** *p* < 0.01). Spearman rank correlation analysis was conducted to evaluate associations between hub gene expression levels and immune cell infiltration scores, with correlations satisfying |ρ| > 0.3 and *p* < 0.05 considered biologically meaningful. Results were visualized as box plots and heatmaps using the ggplot2 and pheatmap packages, respectively.

### 4.8. Metabolic Pathway Activity Analysis

Single-sample gene set enrichment analysis (ssGSEA) was performed to quantify the activity of 86 metabolic pathways in each sample using the GSVA package (version 1.48.0). Gene sets were curated from the Kyoto Encyclopedia of Genes and Genomes (KEGG) and Gene Ontology databases. Pathway enrichment scores were calculated for each sample, and differential pathway activities between the AS and control groups were assessed using the Wilcoxon rank-sum test. Spearman correlation analysis was employed to evaluate associations between hub gene expression and metabolic pathway scores (*p* < 0.05).

### 4.9. Single-Cell RNA Sequencing Analysis

Single-cell RNA-seq data from GSE268839, comprising 3 AS patients and 3 healthy controls, were analyzed using the Seurat package (version 5.3.0). Quality control criteria included: mitochondrial gene percentage < 20%, number of detected genes between 500 and 7000, and RNA molecule counts between 2000 and 25,000. The top 2000 highly variable genes were selected for principal component analysis (PCA). Batch effects were corrected using the Harmony algorithm. Unsupervised clustering was performed using Uniform Manifold Approximation and Projection (UMAP) dimensionality reduction at a resolution of 0.5.

Cell type annotation was performed based on canonical markers, as described in the published literature. The distribution of hub genes across cell types was visualized using FeaturePlot and DotPlot functions. Cell type proportions across samples and conditions were calculated and visualized as stacked bar plots. Metabolic pathway scores in individual cell types were computed using ssGSEA and visualized as heatmaps.

### 4.10. Gene Set Enrichment Analysis

To elucidate the biological functions and signaling pathways associated with hub genes, Gene Set Enrichment Analysis (GSEA) and Gene Set Variation Analysis (GSVA) were conducted using the clusterProfiler package. Gene sets were obtained from the Gene Ontology (GO) and Kyoto Encyclopedia of Genes and Genomes (KEGG) databases. For GSEA, samples were stratified into high and low expression groups based on median hub gene expression. Enrichment scores, normalized enrichment scores (NES), and false discovery rates (FDR) were calculated. Pathways with |NES| > 0.4 and FDR < 0.05 were considered significantly enriched. For GSVA, correlation between hub gene expression and pathway enrichment scores was assessed using Spearman correlation, with statistical significance defined as *p* < 0.05. Upward peaks in GSEA plots indicate pathway enrichment in AS samples with high hub gene expression, while downward deflections indicate enrichment in low-expression samples.

### 4.11. Chromosomal Localization Analysis

To investigate potential co-regulatory mechanisms, chromosomal positions of hub genes were retrieved from the NCBI Gene database. Genomic karyotype maps were generated using the RCircos package (version 1.2.2) to visualize gene distribution across chromosomes.

### 4.12. Construction of the ceRNA Regulatory Network

To elucidate post-transcriptional regulatory mechanisms, a competitive endogenous RNA (ceRNA) network comprising microRNA (miRNA), long non-coding RNA (lncRNA), and messenger RNA (mRNA) interactions was constructed. MiRNA-mRNA interactions were predicted using three databases: miRDB, TargetScan (version 8.0), and miRTarBase (version 9.0), with only interactions validated in at least two databases retained. LncRNA-miRNA interactions were predicted using starBase v3.0 and LncBase v3.0. The network was visualized using Cytoscape software (version 3.10.0), with nodes representing mRNAs, miRNAs, and lncRNAs, and edges representing regulatory interactions. Network topology parameters including degree centrality and betweenness centrality were calculated to identify hub regulators.

### 4.13. Natural Compound Library Construction and Binding Prediction

Traditional Chinese medicines (TCMs) commonly used for AS treatment were identified based on published literature. Chemical components were retrieved from the Traditional Chinese Medicine Systems Pharmacology Database (TCMSP, https://www.tcmsp-e.com/tcmsp.php; accessed on 12 December 2025) and BATMAN-TCM (http://bionet.ncpsb.org/batman-tcm/; accessed on 12 December 2025). Because most AS-treating TCMs are used topically, no oral bioavailability (OB) or drug-likeness (DL) filters were applied. Drug-target interaction (DTI) prediction was performed using the DeepPurpose framework, an open-source deep learning library that implements an encoder–decoder architecture for compound-protein binding affinity prediction [25]. DeepPurpose takes as input the simplified molecular-input line-entry system (SMILES) string of each compound and the amino acid sequence of each target protein, encodes them into high-dimensional vector representations via independent molecular encoders, concatenates the learned embeddings, and feeds them into a multi-layer perceptron (MLP) decoder to predict binding affinity scores. The library supports 8 compound encoders and 7 protein encoders, enabling over 50 encoder–decoder combinations. In this study, compound SMILES were retrieved from PubChem based on compound identifiers (CIDs), and target protein sequences for the six hub gene products were obtained from UniProt.

Binding affinity scores were predicted for all 16,110 compound-target pairs (2685 compounds × 6 hub gene targets). The total binding score for each compound was computed by summing the predicted scores across all six targets. The top 20 compounds ranked by total binding score were visualized using a lollipop plot generated with R (version 4.3.0). These predictions represent computational estimates of binding potential and should be interpreted as hypothesis-generating; experimental validation through in vitro binding assays is required to confirm the predicted interactions.

### 4.14. Statistical Analysis

All statistical analyses were performed using R software (version 4.3.0). Continuous variables were compared using Student’s t-test for normally distributed data or the Wilcoxon rank-sum test for non-normally distributed data. Categorical variables were analyzed using Fisher’s exact test or the Chi-square test as appropriate. Correlation analyses were conducted using Spearman’s rank correlation coefficient. A two-tailed *p*-value < 0.05 was considered statistically significant. Multiple testing correction was applied using the Benjamini–Hochberg method where appropriate.

Benjamini–Hochberg FDR correction was applied in the following analyses: (i) differential expression analysis (adjusted *p*-values reported as adj.P.Val); (ii) Gene Ontology enrichment analysis (FDR < 0.05); and (iii) Gene Set Enrichment Analysis (FDR < 0.05). Uncorrected *p*-values were used for: (i) WGCNA module–trait correlations (Pearson correlation, *p* < 0.05); (ii) CIBERSORT immune cell comparisons (Wilcoxon rank-sum test, *p* < 0.05); (iii) Spearman correlation analyses between hub genes and immune/metabolic scores (*p* < 0.05); and (iv) ssGSEA pathway comparisons (Wilcoxon rank-sum test, *p* < 0.05).

## 5. Conclusions

In conclusion, this study provides a comprehensive multi-omics characterization of ankylosing spondylitis (AS) that highlights coordinated alterations in energy-metabolism pathways alongside changes in peripheral immune-cell composition. Our analyses revealed a profound, simultaneous downregulation of oxidative phosphorylation, the TCA cycle, and glycolysis, which indicates the altered immune landscape, most notably the observed depletion of CD8+ T cells and expansion of neutrophils. We identified a six-gene signature (*MFN2*, *SLC27A3*, *SMG7*, *RHOB*, *AKR1B1*, *LCOR*) that may represent a transcriptomic link between metabolic alterations and immune-associated changes in AS. Regulation of these genes appears to involve multiple layers: *MFN2*, *SLC27A3*, and *SMG7* are co-located on chromosome 1, raising the possibility of shared cis-regulatory influence, and ceRNA network analysis identified competing endogenous RNA interactions that may modulate their post-transcriptional expression. These observations suggest a degree of regulatory coordination, although the functional significance of this architecture in AS remains to be established experimentally.

From a translational perspective, the six-gene signature was used to develop a diagnostic model that demonstrated moderate cross-validated performance and preliminary cross-cohort reproducibility through leave-one-dataset-out validation between two whole-blood cohorts, though further validation in independent, larger cohorts remains necessary. Deep learning-based compound screening prioritized natural compounds such as betaine as candidates for experimental validation against the hub gene network. Collectively, this work not only provides a foundation for developing metabolism-targeted diagnostics and therapies but also outlines a path for future research to validate these mechanisms and translate them into clinical applications for ankylosing spondylitis.

## Data Availability

All datasets analyzed in this study are publicly available from the NCBI Gene Expression Omnibus (GEO): GSE25101, GSE73754, GSE11886, and GSE268839. The traditional Chinese medicine compound library was compiled from TCMSP (https://www.tcmsp-e.com/tcmsp.php; accessed on 12 December 2025) and BATMAN-TCM (http://bionet.ncpsb.org/batman-tcm/; accessed on 12 December 2025). Drug-target interaction prediction was performed using the DeepPurpose framework (https://github.com/kexinhuang12345/DeepPurpose; accessed on 15 December 2025). The analysis code and parameter settings are available from the corresponding author upon reasonable request.

## Supplementary Materials

The following supporting information can be downloaded at: https://www.mdpi.com/article/10.3390/ijms27093860/s1.
