# Supplementary material for: Multi-Omics Integration Identifies a Six-Gene Diagnostic Signature for Ankylosing Spondylitis via Metabolic–Immune Crosstalk
Source: Int J Mol Sci. 2026 Apr 27;27(9):3860. doi: 10.3390/ijms27093860 (PMC13164271; doi:10.3390/ijms27093860)
Supplement: Supplementary file 1 [file ijms-27-03860-s001.zip › 0415Supplementary Figures.pdf]

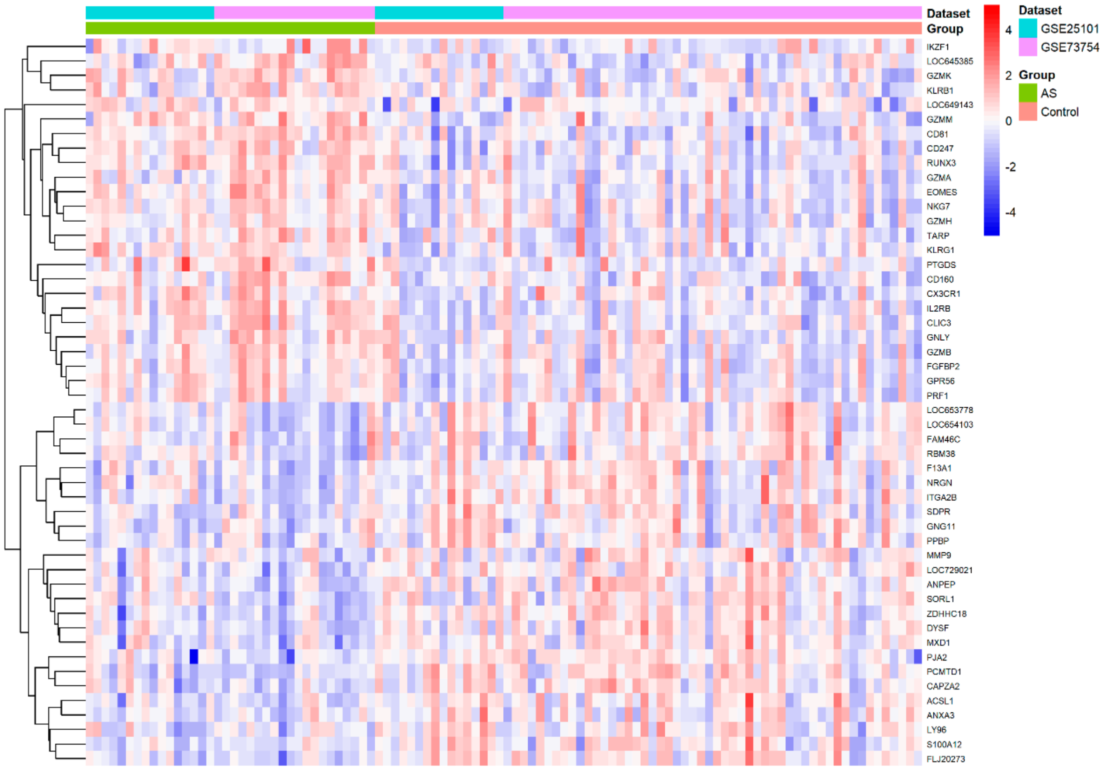

Figure S1 DEG Heatmap

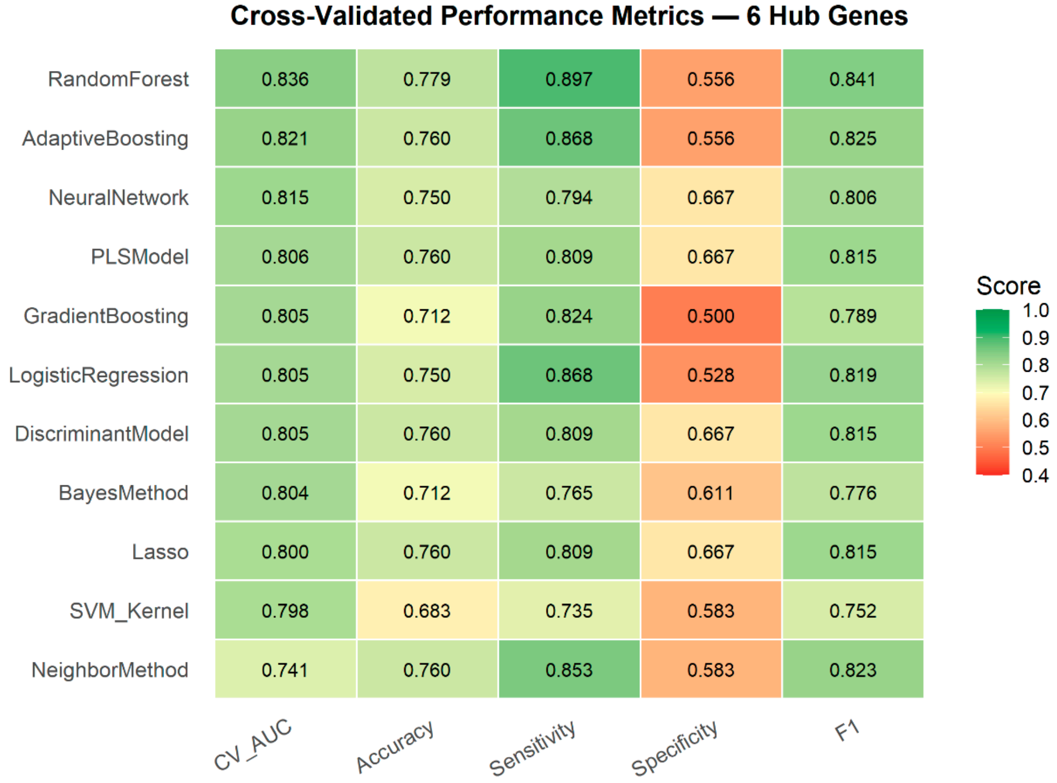

Figure S2: Cross-validated performance metrics heatmap for 11 machine learning algorithms using the six-gene signature. Each row represents one algorithm; columns display five evaluation metrics: CV AUC, Accuracy, Sensitivity, Specificity, and F1-score, all derived from 5-fold cross-validated out-of-fold predictions on

the merged whole-blood cohort (n=104). Cell values indicate metric scores, with color gradient (red–yellow–green) facilitating visual comparison. RandomForest achieved the highest CV AUC (0.836), while Specificity ranged from 0.500 (NeighborMethod) to 0.667 (multiple algorithms), consistent with the moderate effect sizes expected in blood-based transcriptomic biomarker studies. All preprocessing steps were embedded within cross-validation folds to prevent information leakage.

Round 1: Train = GSE25101 (n=32), Validate = GSE73754 (n=72)
Round 2: Train = GSE73754 (n=72), Validate = GSE25101 (n=32)
95% CI calculated by bootstrap (n=2000). Ordered by Mean Validation AUC.

Algorithm	R1 Val AUC	R1 Acc	R1 Sens	R1 Spec	R2 Val AUC	R2 Acc	R2 Sens	R2 Spec	Mean AUC
SVM_Kernel	0.861 (0.767–0.938)	0.736	0.692	0.850	0.676 (0.480–0.848)	0.625	1.000	0.250	0.768
NeuralNetwork	0.834 (0.719–0.925)	0.736	0.692	0.850	0.668 (0.465–0.863)	0.500	0.875	0.125	0.751
GradientBoosting	0.778 (0.655–0.889)	0.708	0.692	0.750	0.715 (0.512–0.906)	0.562	0.875	0.250	0.746
AdaptiveBoosting	0.784 (0.665–0.889)	0.750	0.769	0.700	0.699 (0.500–0.883)	0.500	0.875	0.125	0.742
RandomForest	0.813 (0.693–0.911)	0.694	0.673	0.750	0.664 (0.453–0.855)	0.469	0.875	0.062	0.738
BayesMethod	0.822 (0.703–0.919)	0.722	0.692	0.800	0.637 (0.430–0.820)	0.625	0.875	0.375	0.730
NeighborMethod	0.810 (0.689–0.910)	0.694	0.635	0.850	0.600 (0.395–0.783)	0.469	0.812	0.125	0.705
LogisticRegression	0.772 (0.656–0.878)	0.681	0.654	0.750	0.617 (0.410–0.812)	0.531	1.000	0.062	0.695
Lasso	0.772 (0.654–0.880)	0.681	0.654	0.750	0.609 (0.410–0.801)	0.469	0.875	0.062	0.691
DiscriminantModel	0.784 (0.654–0.896)	0.653	0.596	0.800	0.594 (0.375–0.797)	0.625	1.000	0.250	0.689
PLSModel	0.783 (0.651–0.893)	0.653	0.596	0.800	0.594 (0.387–0.805)	0.594	1.000	0.188	0.689

Figure S3. Leave-one-dataset-out(LODO) validation performance across 11 algorithms. Summary of validation metrics from two LODO rounds using tissue-matched whole-blood data. Round 1 (train: GSE25101, n=32; validate: GSE73754, n=72): SVM_Kernel achieved the highest validation AUC of 0.861 (95% CI: 0.767–0.938), with all algorithms exceeding 0.77. Round 2 (train: GSE73754, n=72; validate: GSE25101, n=32): GradientBoosting achieved the highest validation AUC of 0.715 (95% CI: 0.512–0.906). The wider confidence intervals and lower overall performance in Round 2 reflect the smaller validation sample size and associated class imbalance. Batch correction (ComBat) was applied to the merged dataset prior to LODO splitting; consequently, these results should be interpreted as estimates of cross-dataset reproducibility rather than strict independent validation.

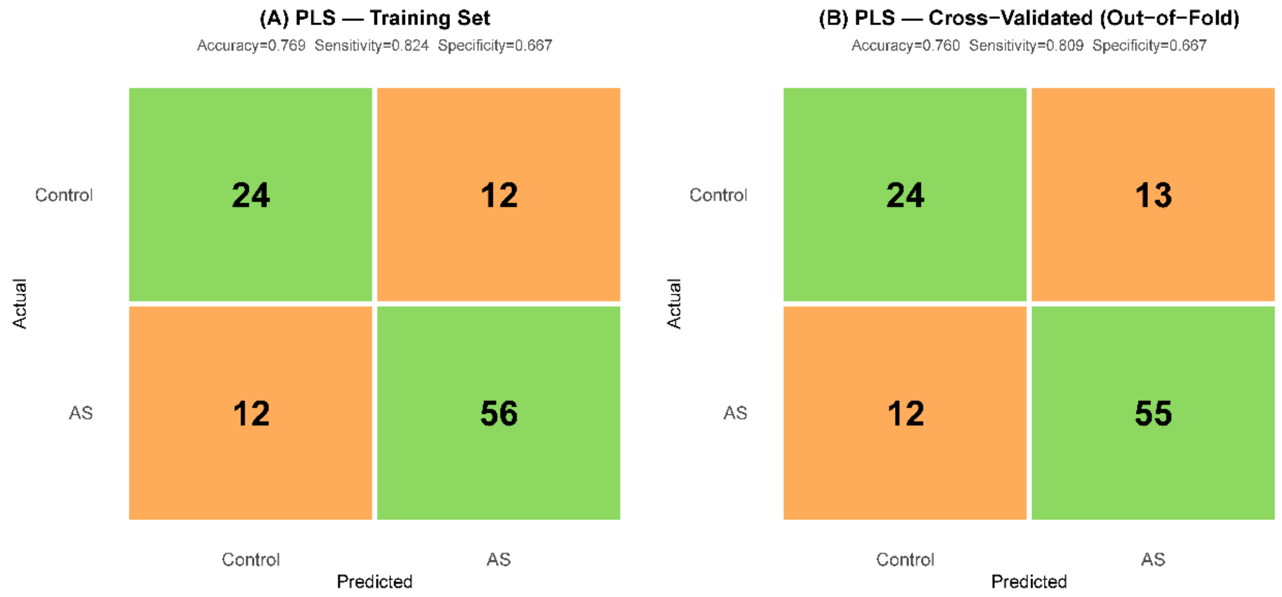

Figure S4. Confusion matrices of the Partial Least Squares (PLS) model trained on all 16 metabolism-related candidate genes for SHAP-based feature importance analysis. (A) Training set confusion matrix (n=104). **(B)** Cross-validated out-of-fold confusion matrix. The high concordance between panels reflects the regularized, low-complexity nature of the PLS model (ncomp=5), indicating minimal overfitting.

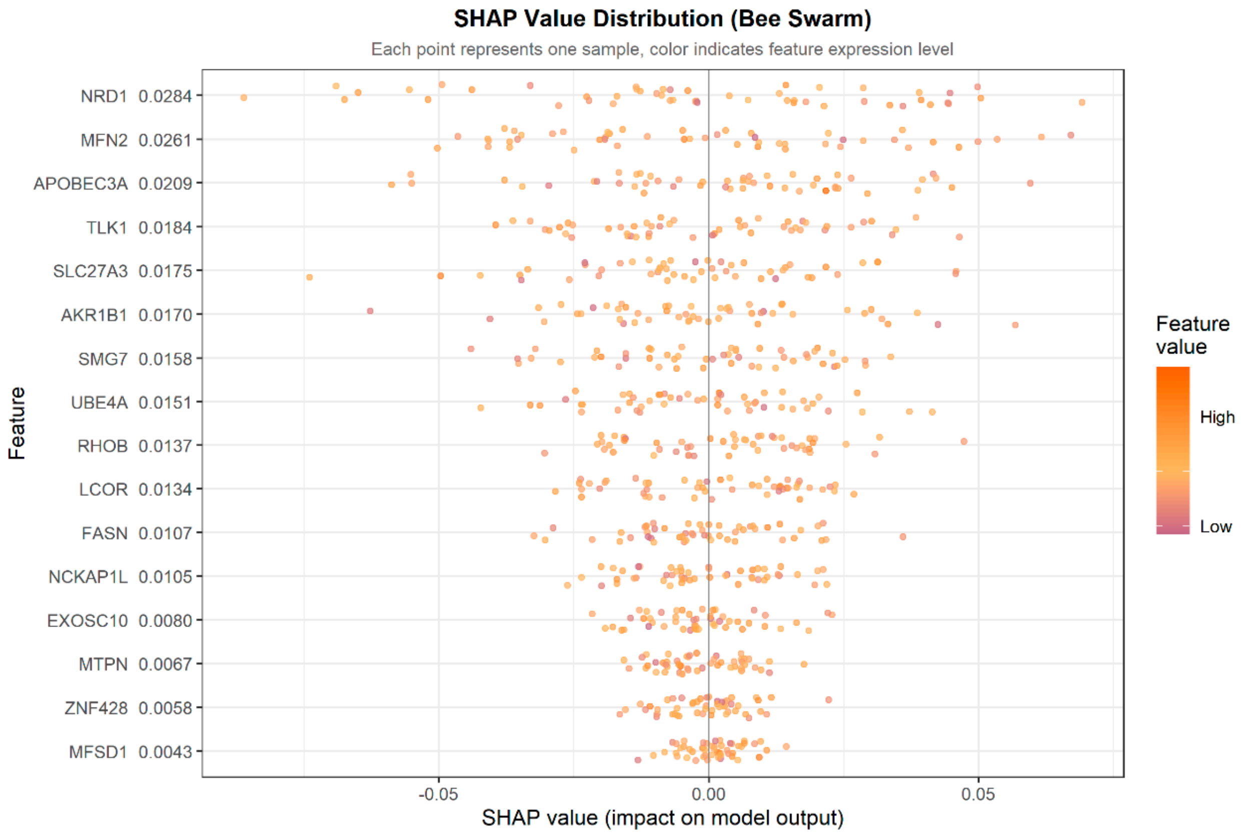

Figure S5. SHAP bee swarm plot displaying feature value distribution and prediction impact for all 16

metabolism-related candidate genes. Each point represents one sample, with horizontal position indicating SHAP value (impact on model output) and color indicating feature expression level (yellow/orange = high, purple = low). Features are ranked by mean |SHAP| value: *NRD1* (0.028) and *MFN2* (0.026) occupy the top two positions, followed by APOBEC3A (0.021), TLK1 (0.018), *SLC27A3* (0.018), *AKR1B1* (0.017), *SMG7* (0.016), UBE4A (0.015), *RHOB* (0.014), and *LCOR* (0.013). All six hub genes (*MFN2*, *SLC27A3*, *AKR1B1*, *SMG7*, *RHOB*, *LCOR*) are confirmed within the top 10 features. *MFN2* shows the strongest directional effect, with high expression (yellow) associated with negative SHAP values (toward Control classification), consistent with its downregulation in AS patients.

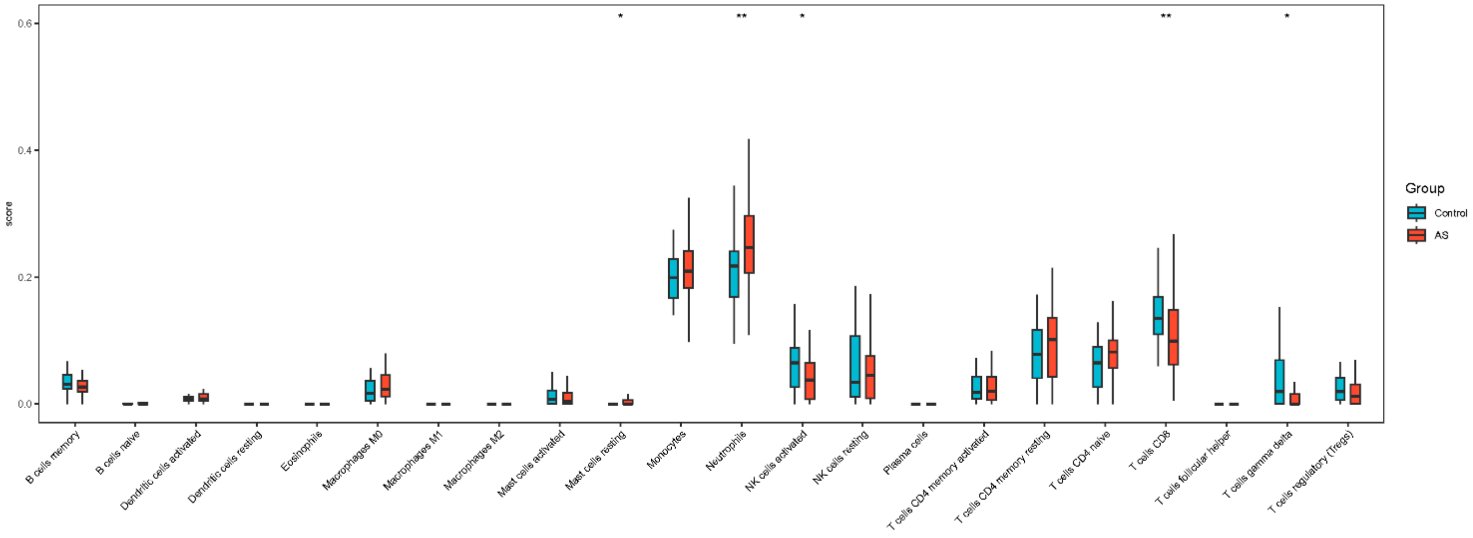

Figure S6. CIBERSORT-based immune cell infiltration comparison between AS patients and healthy controls. Box plots comparing the relative proportions of 22 immune cell types. Key differences include significantly decreased CD8+ T cells (**P < 0.01), elevated CD4+ memory-activated T cells (*P < 0.05), and reduced CD4+ memory-resting T cells (*P < 0.05).

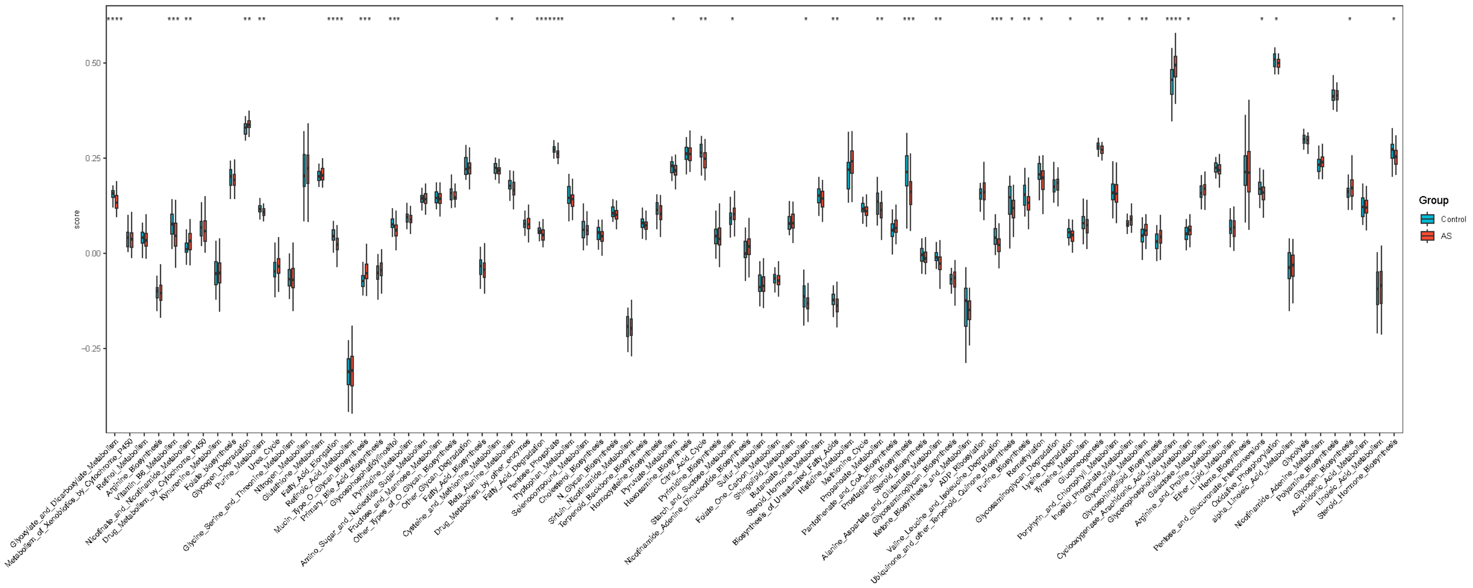

Figure S7. ssGSEA-based metabolic pathway activity comparison between AS patients and healthy controls. Box plots comparing the enrichment scores of 86 metabolic pathways. Core energy metabolism pathways—oxidative phosphorylation, TCA cycle, and glycolysis—are downregulated (P < 0.01), while glyoxylate and glutathione metabolism are upregulated (P < 0.001).

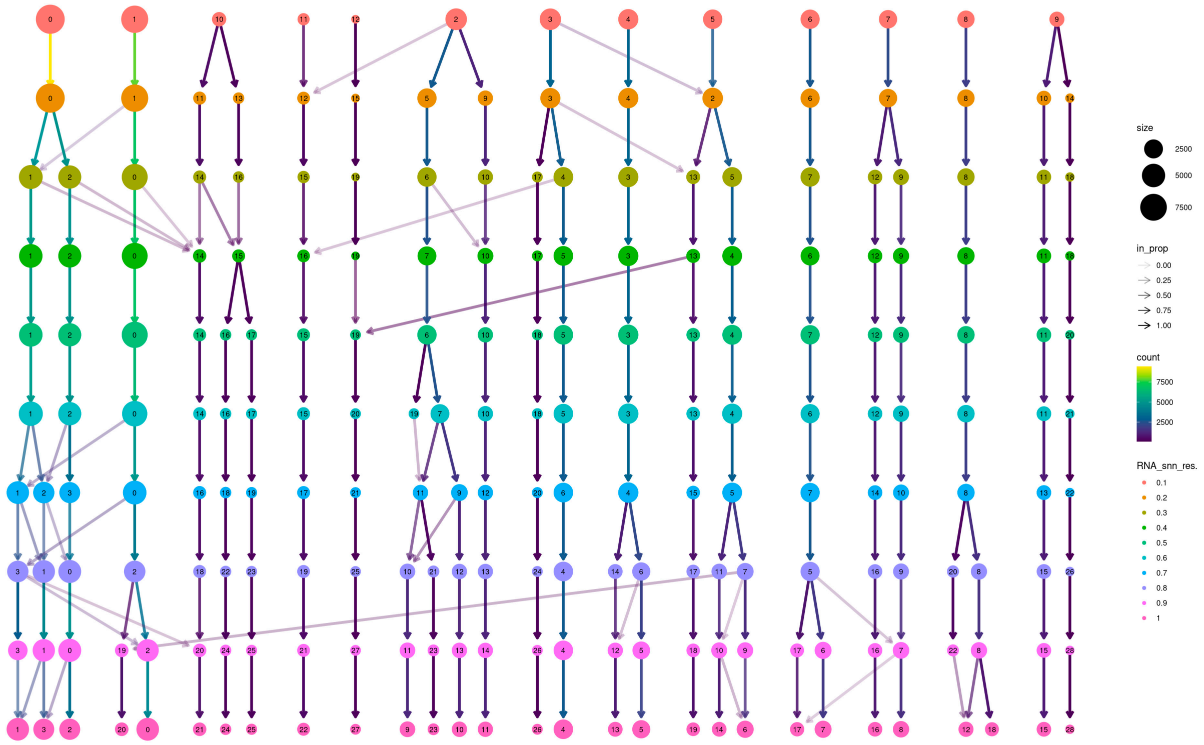

Figure S8. Clustering tree showing hierarchical relationships between cell clusters at different resolutions.

Directed graph displaying cluster relationships across resolution parameters from 0.1 to 1.0. Node size represents cluster size (2,500-7,500 cells), and node color indicates resolution parameter. Arrows show cluster splitting patterns as resolution increases. The selected resolution of 0.5 (green nodes) balances biological resolution and annotation consistency.

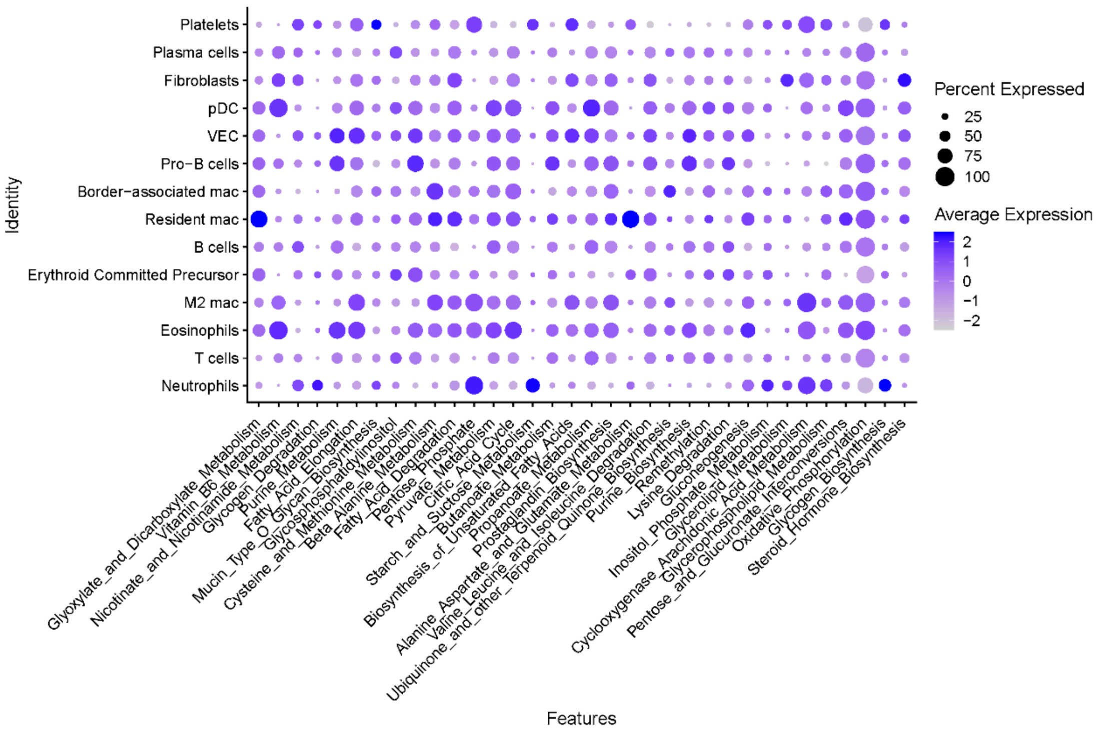

Figure S9. Dot plot of metabolic pathway enrichment scores across 14 cell types. Color intensity represents average expression z-score, and dot size indicates the percentage of cells with pathway activity. Neutrophils show strong glycolytic pathway activity, T cells exhibit high oxidative phosphorylation, and macrophages display enhanced fatty acid metabolism, revealing cell type-specific metabolic preferences in AS pathogenesis."

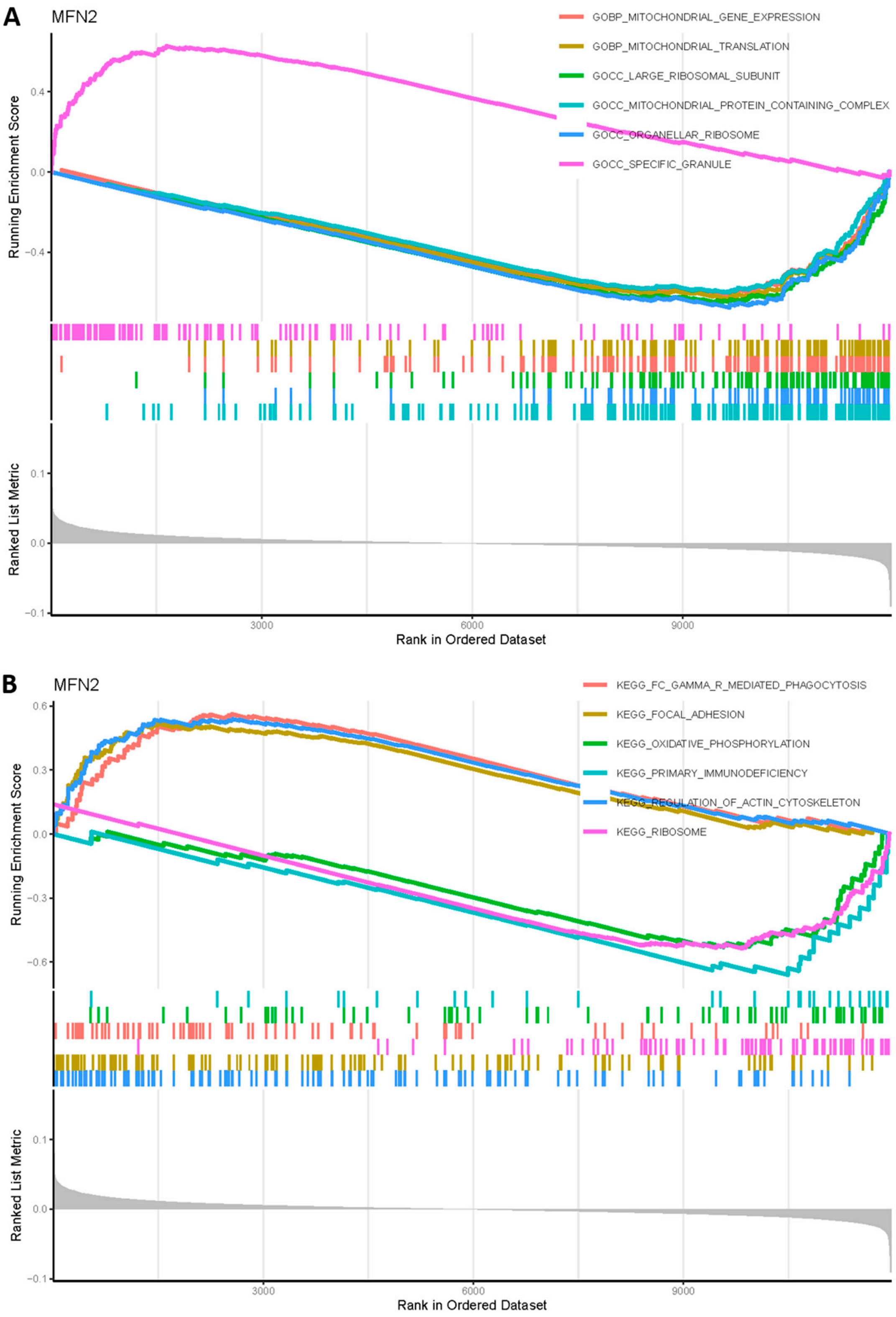

Figure S10. GSEA enrichment plots for *MFN2*. (A) GO enrichment showing positive enrichment in mitochondrial gene expression, mitochondrial translation, and mitochondrial protein-containing complexes. (B) KEGG pathway enrichment displaying associations with oxidative phosphorylation, ribosome, and actin cytoskeleton regulation.

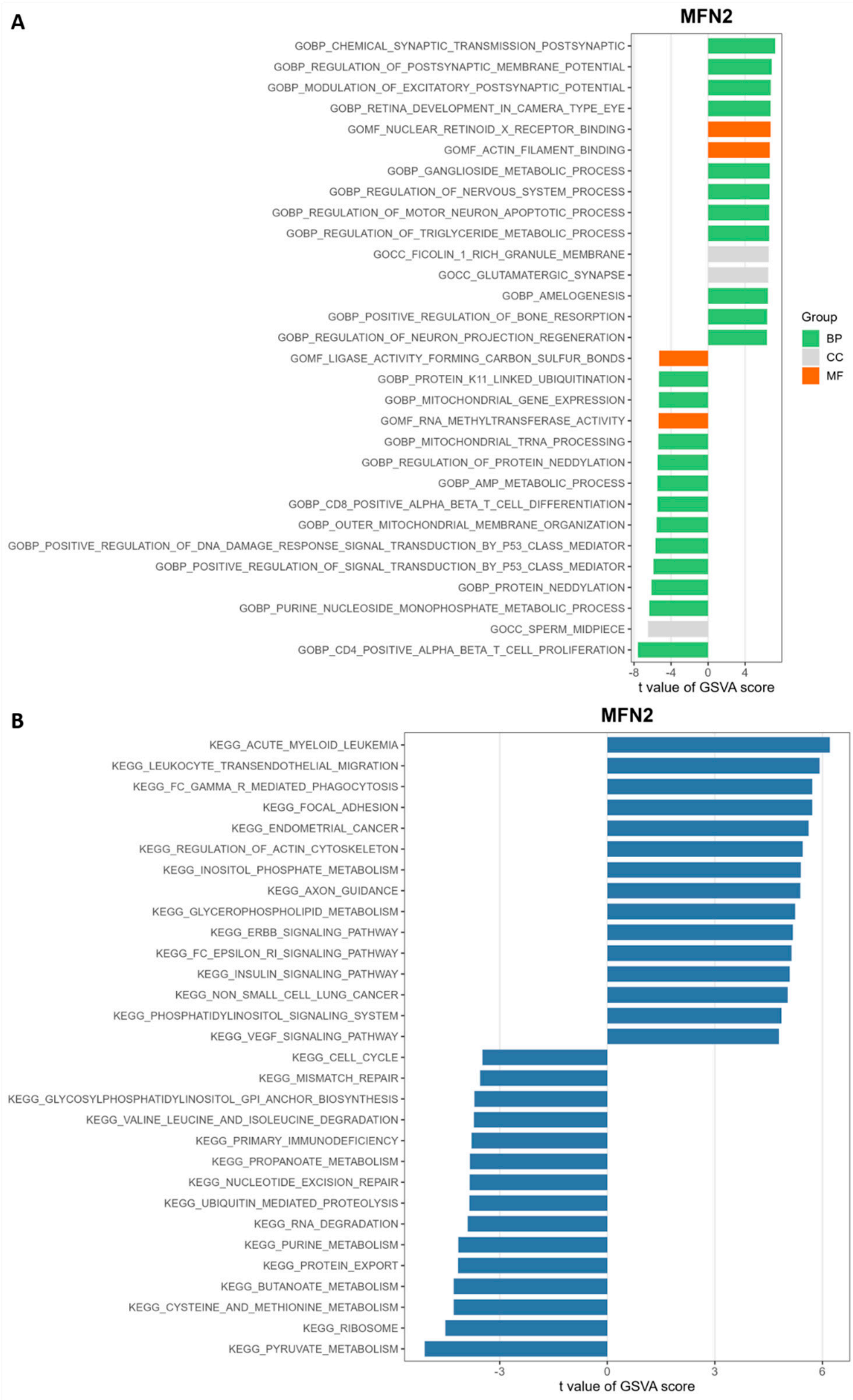

Figure S11. GSVA-GO and KEGG correlation bar plots for *MFN2*. (A) *MFN2* shows positive correlations with chemical synaptic transmission, regulation of postsynaptic membrane potential, modulation of excitatory postsynaptic potential, and neuronal developmental processes, while negative correlations were observed for ligase activity forming carbon-sulfur bonds, RNA methyltransferase activity, outer mitochondrial membrane organization, and p53 class mediator-dependent signaling. **(B)** *MFN2* positively correlates with acute myeloid

leukemia, leukocyte transendothelial migration, Fc gamma R-mediated phagocytosis, focal adhesion, and regulation of actin cytoskeleton, whereas pyruvate metabolism, ribosome, protein export, RNA degradation, and ubiquitin-mediated proteolysis were negatively correlated.

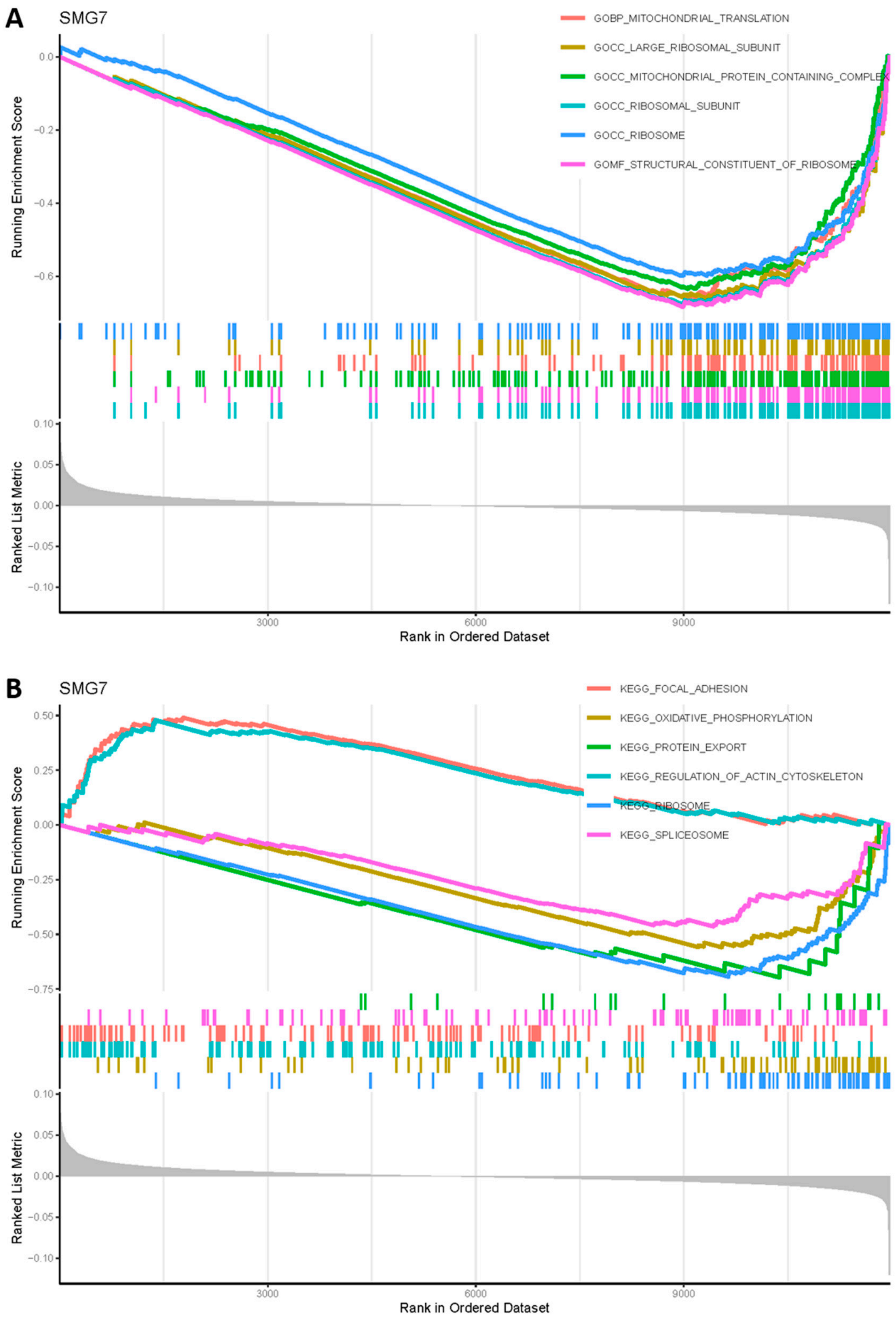

Figure S12. GSEA enrichment plots for *SMG7*. (A) GO enrichment showing negative enrichment in

mitochondrial translation, large ribosomal subunit, mitochondrial protein-containing complex, ribosomal subunit, ribosome, and structural constituent of ribosome. **(B)** KEGG pathway enrichment displaying positive enrichment in focal adhesion and regulation of actin cytoskeleton, while oxidative phosphorylation, ribosome, spliceosome, and protein export showed negative enrichment.

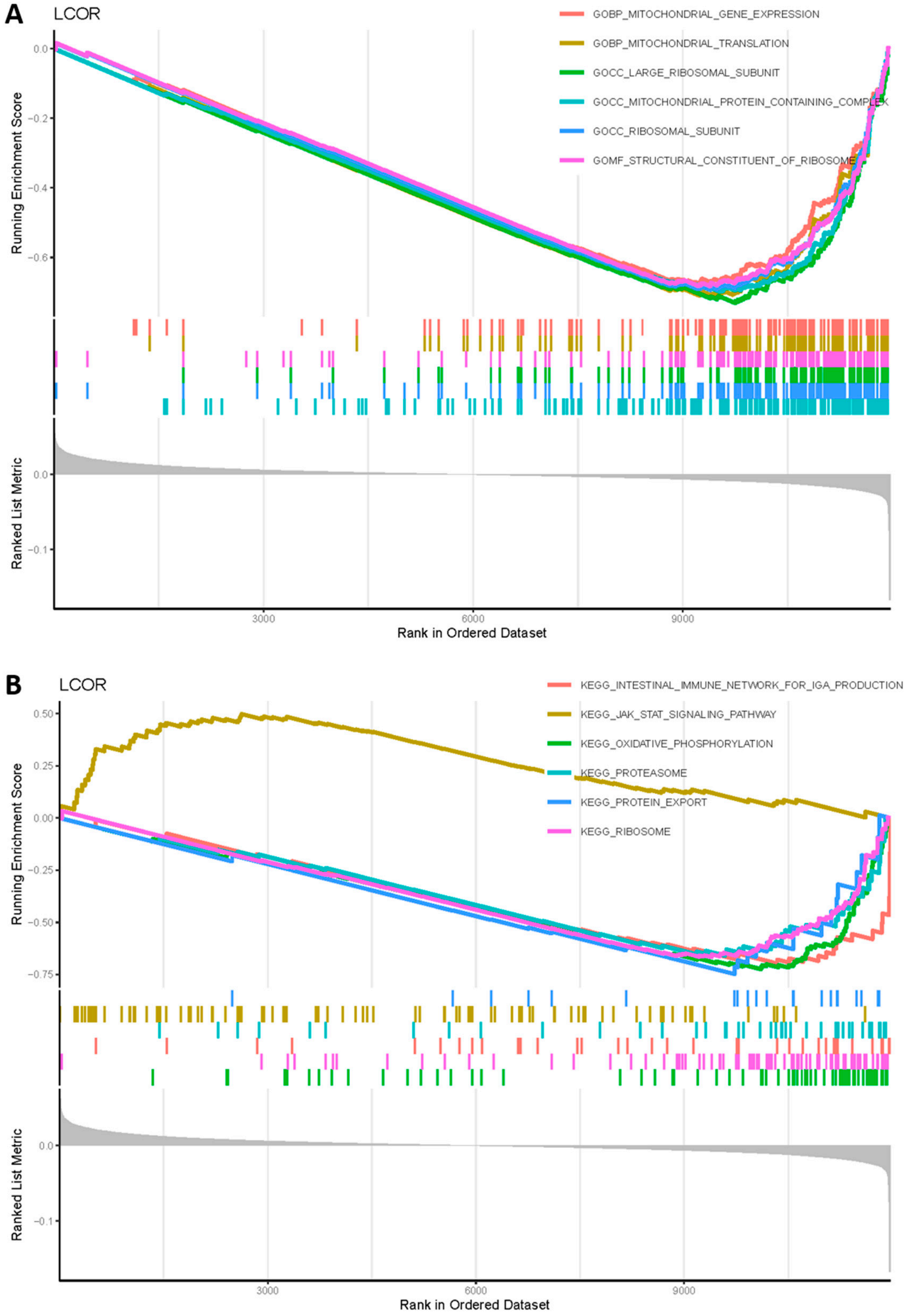

Figure S13. GSEA enrichment plots for *LCOR*. (A) GO enrichment showing negative enrichment in mitochondrial gene expression, mitochondrial translation, large ribosomal subunit, mitochondrial protein-containing complex, ribosomal subunit, and structural constituent of ribosome. **(B)** KEGG pathway enrichment displaying association with JAK-STAT signaling pathway, while intestinal immune network for IgA production, oxidative phosphorylation, proteasome, protein export, and ribosome showed negative enrichment.

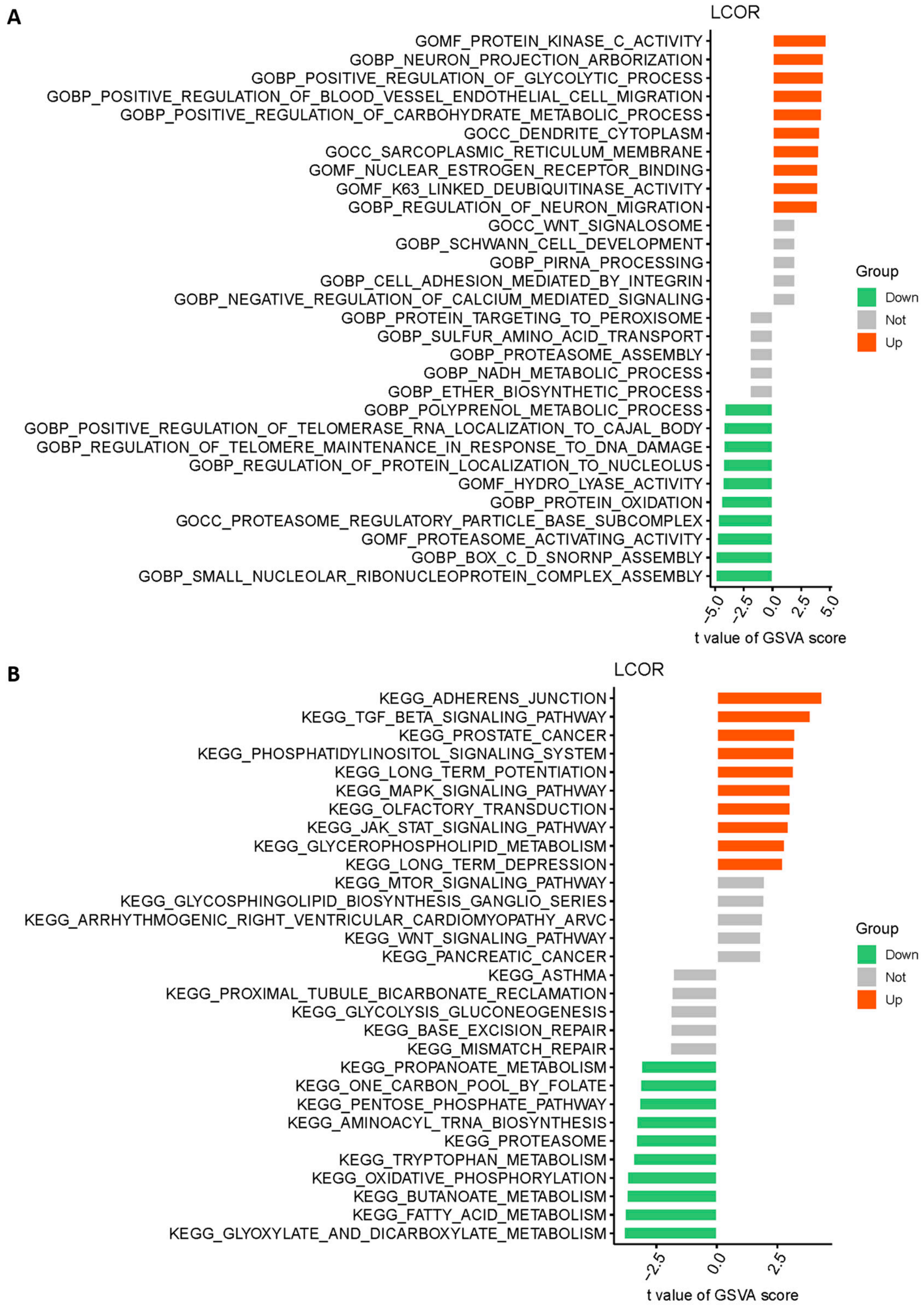

Figure S14. GSVA-GO and KEGG correlation bar plots for *LCOR*. (A) *LCOR* positively correlated with glycolytic process, endothelial cell migration, and neuron migration-related GO terms, whereas negatively correlated terms were mainly associated with telomere maintenance, nucleolar protein localization, and proteasome-related functions. **(B)** KEGG analysis showed positive correlations with adherens junction, TGF-β, MAPK, and JAK-STAT signaling, while negatively correlated pathways were primarily enriched in oxidative

phosphorylation, fatty acid metabolism, pentose phosphate pathway, and proteasome.

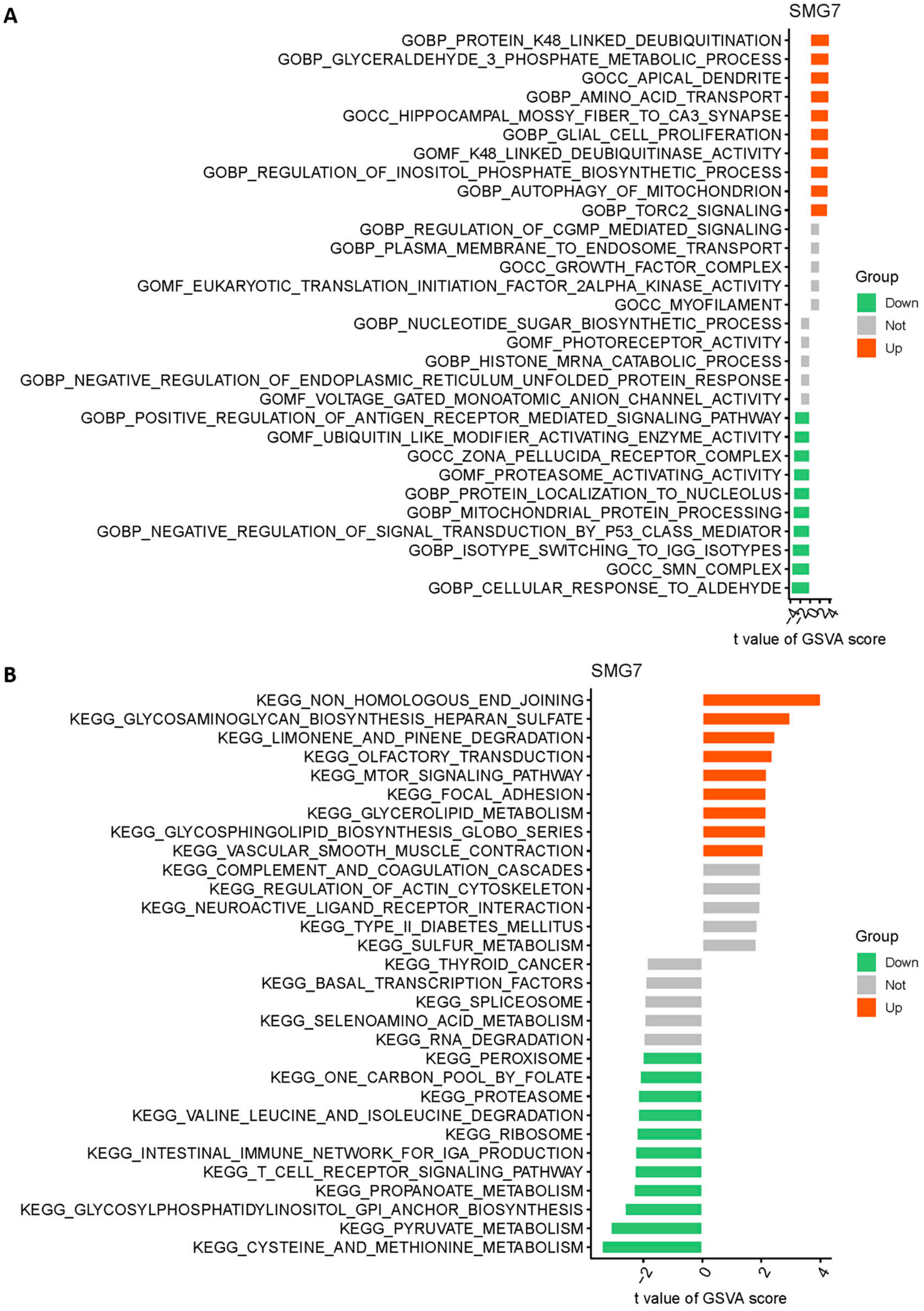

Figure S15. GSVA-GO and KEGG correlation bar plots for *SMG7*. (A) *SMG7* positively correlated with K48-linked deubiquitination, amino acid transport, mitochondrial autophagy, and TORC2 signaling, whereas negatively correlated GO terms were enriched in antigen receptor-mediated signaling, proteasome-related

activity, and mitochondrial protein processing. **(B)** KEGG analysis showed positive correlations with non-homologous end joining, mTOR signaling, and focal adhesion, while negatively correlated pathways were mainly related to proteasome, ribosome, T-cell receptor signaling, and pyruvate metabolism.

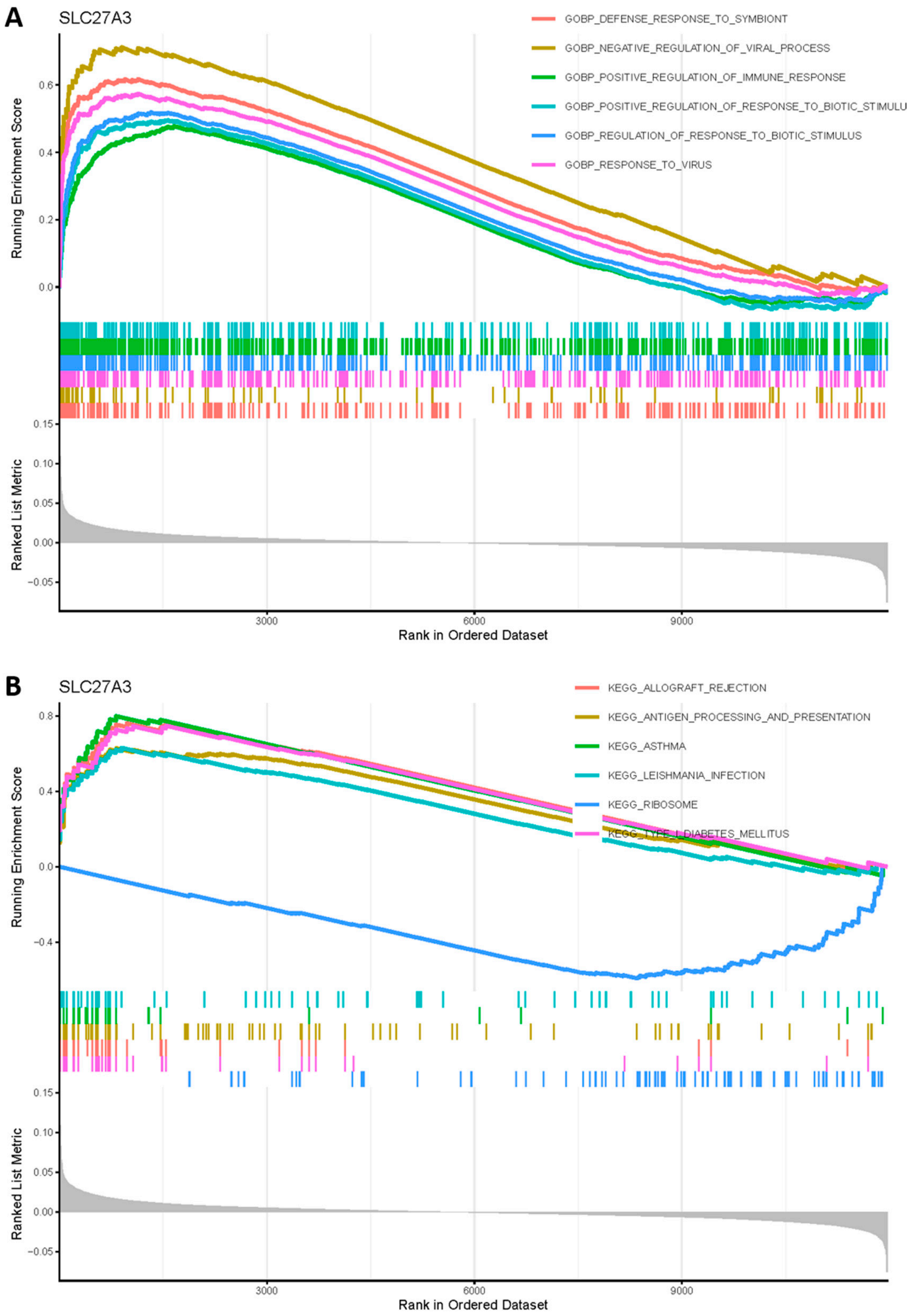

Figure S16. GSEA enrichment plots for *SLC27A3*. (A) GO enrichment showing positive enrichment in defense

response to symbiont, negative regulation of viral process, positive regulation of immune response, positive regulation of response to biotic stimulus, regulation of response to biotic stimulus, and response to virus. **(B)** KEGG pathway enrichment displaying associations with allograft rejection, antigen processing and presentation, asthma, leishmania infection, and type I diabetes mellitus, while ribosome showed negative enrichment.

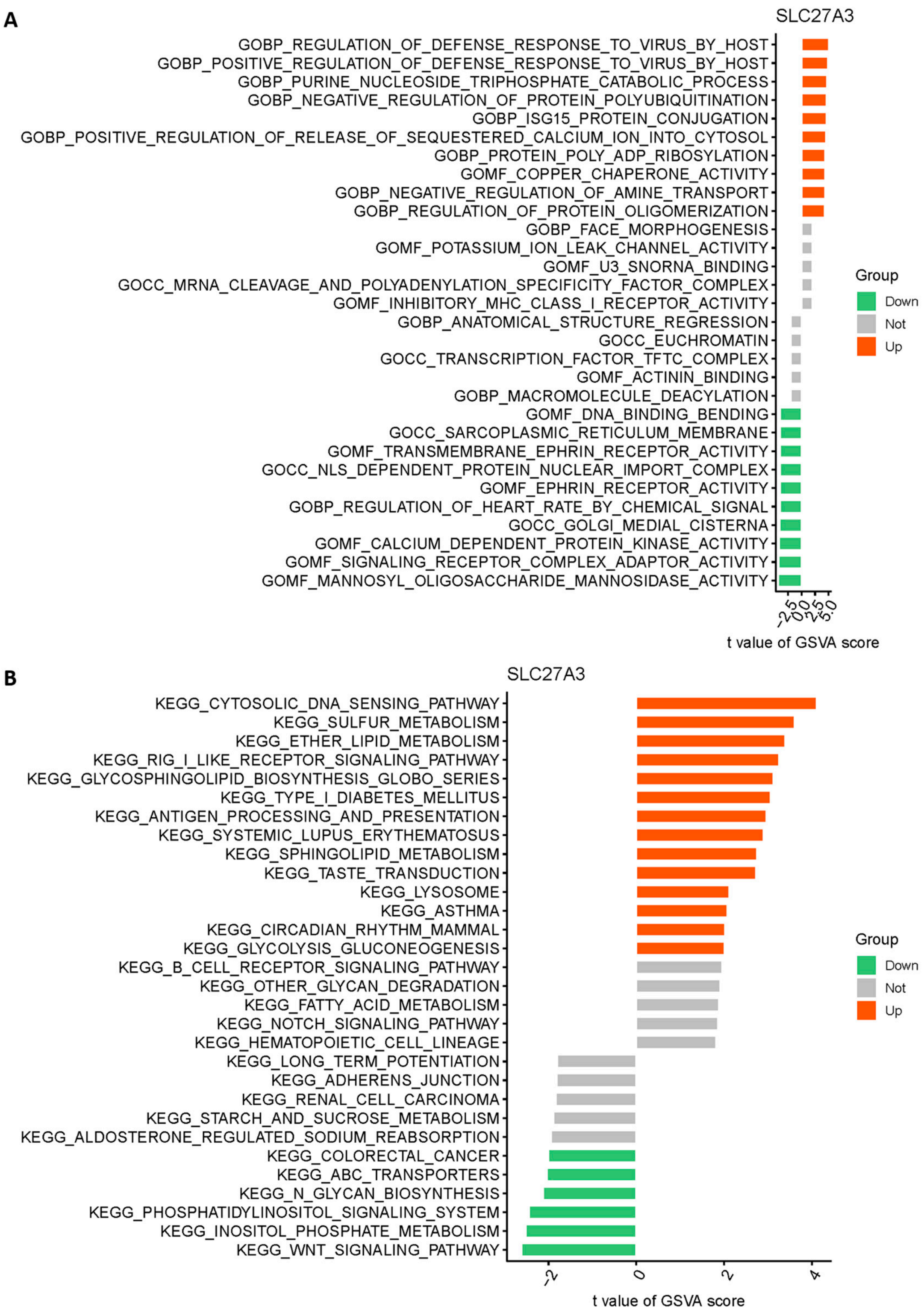

Figure S17. GSVA-GO and KEGG correlation bar plots for *SLC27A3*. (A) *SLC27A3* shows positive correlations with ISG15 conjugation, protein poly-ADP-ribosylation, and defense response to virus regulation. **(B)** *SLC27A3* positively correlates with cytosolic DNA-sensing pathway, sulfur metabolism, ether lipid metabolism, and RIG-I-like receptor signaling pathway, while Wnt signaling pathway, inositol phosphate metabolism, and phosphatidylinositol signaling system were negatively correlated.

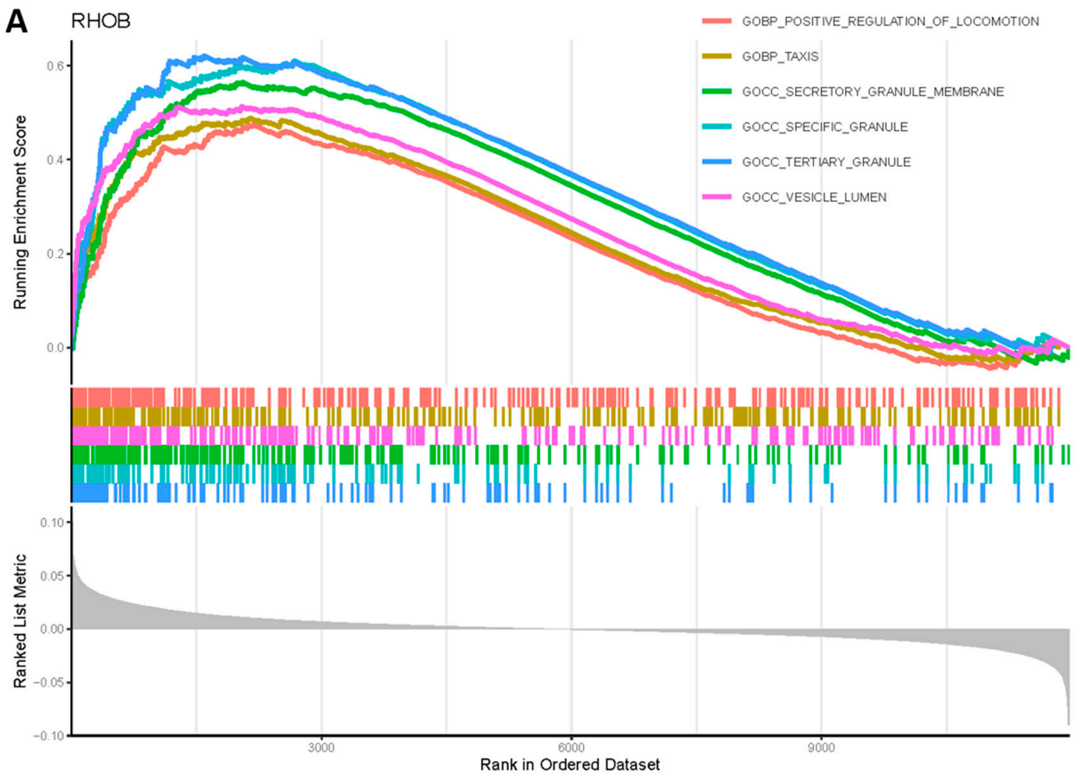

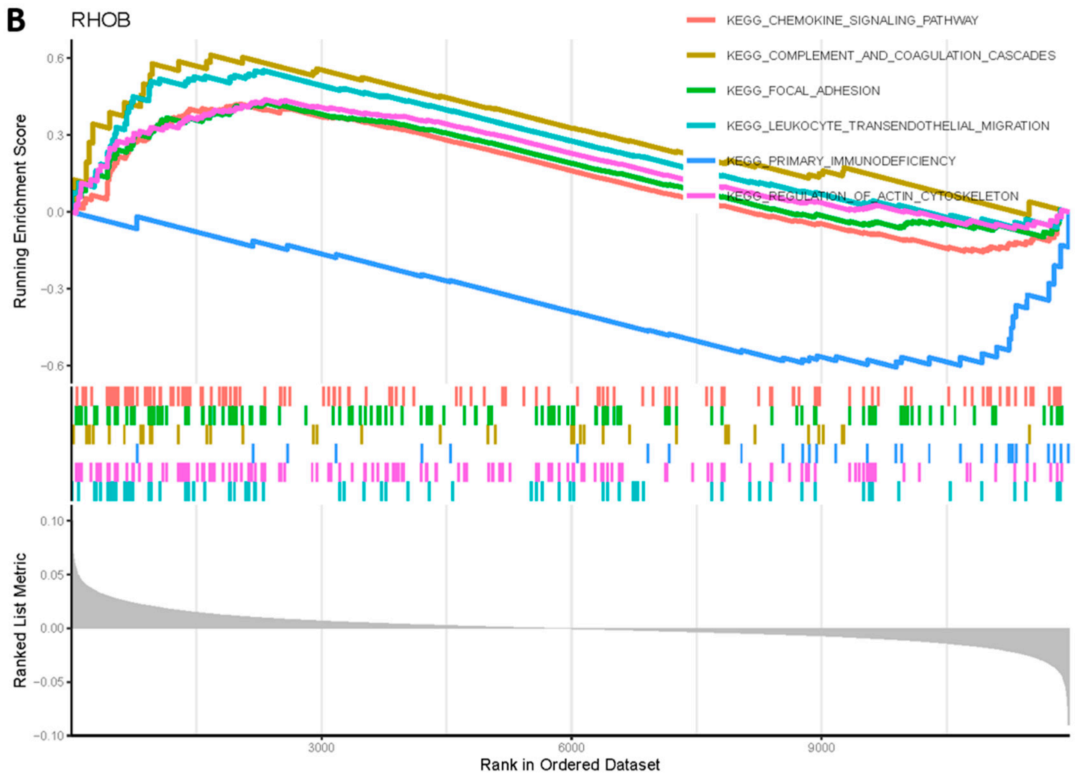

Figure S18. GSEA enrichment plots for *RHOB*. (A) GO enrichment showing positive regulation of locomotion, taxis, and granule-related cellular components. **(B)** KEGG pathway enrichment displaying associations with chemokine signaling, leukocyte transendothelial migration, and actin cytoskeleton regulation.

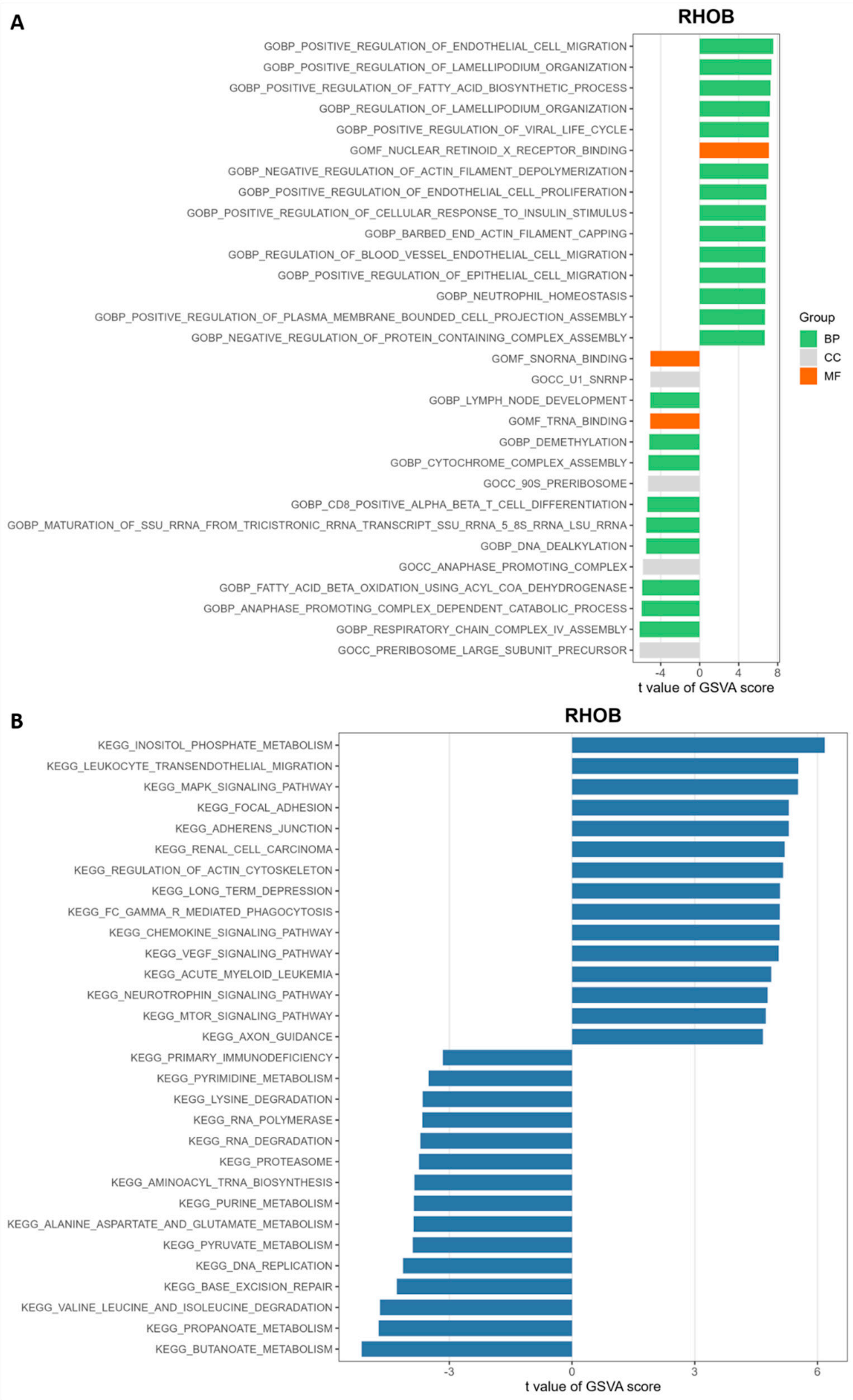

Figure S19. GSVA-GO and KEGG correlation bar plots for *RHOB*. (A) *RHOB* shows positive correlations with positive regulation of endothelial cell migration, lamellipodium organization, positive regulation of fatty acid biosynthetic process, positive regulation of endothelial cell proliferation, epithelial cell migration, and neutrophil homeostasis, while negative correlations were observed for snRNA binding, tRNA binding, DNA dealkylation, cytochrome complex assembly, and respiratory chain complex IV assembly. **(B)** *RHOB* positively

correlates with inositol phosphate metabolism, leukocyte transendothelial migration, MAPK signaling pathway, focal adhesion, adherens junction, regulation of actin cytoskeleton, chemokine signaling pathway, and VEGF signaling pathway, whereas butanoate metabolism, propanoate metabolism, valine, leucine and isoleucine degradation, base excision repair, DNA replication, pyruvate metabolism, and RNA degradation were negatively correlated.

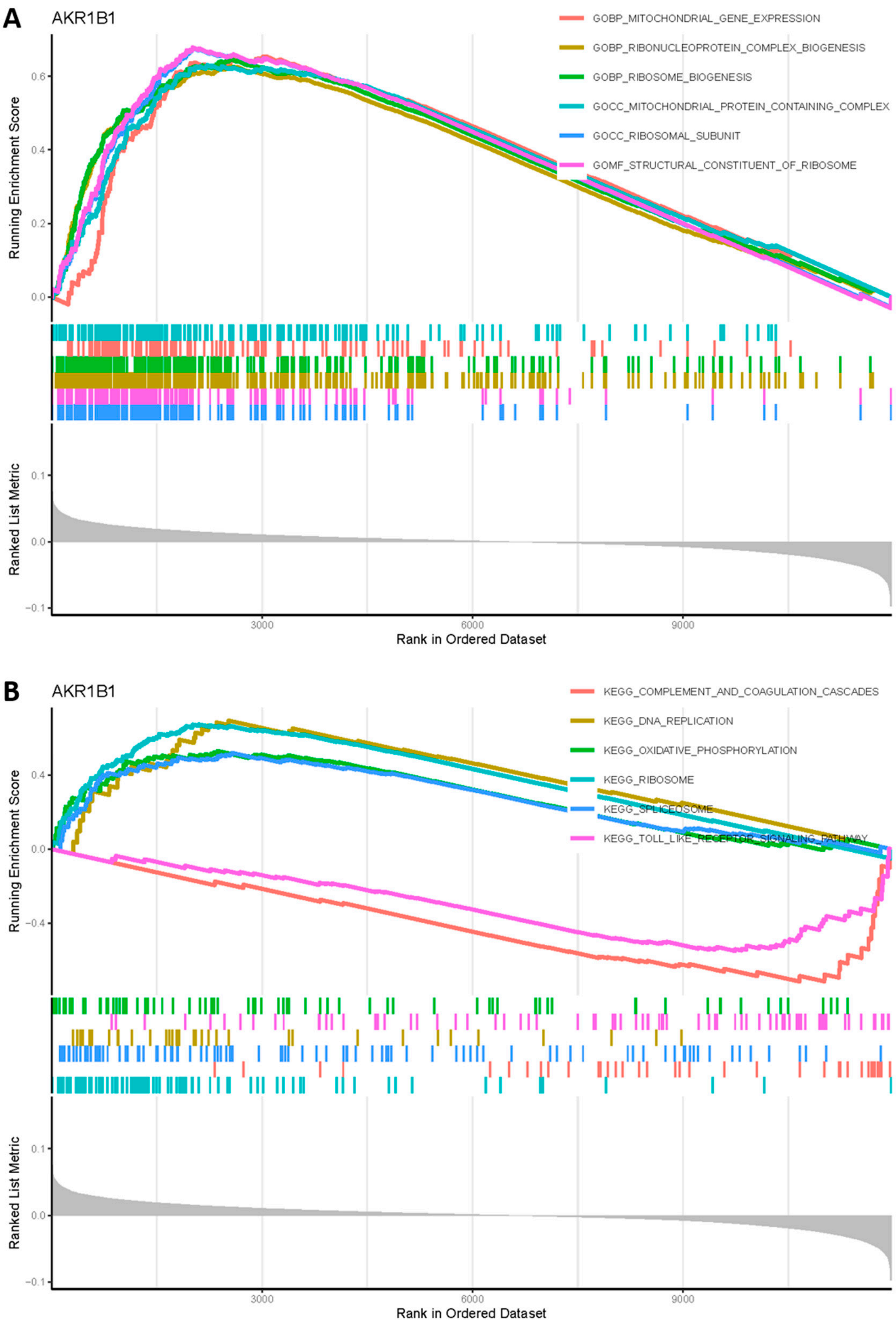

Figure S20. GSEA enrichment plots for *AKR1B1*. (A) GO enrichment showing positive enrichment in mitochondrial gene expression, ribosome biogenesis, and mitochondrial protein complexes. **(B)** KEGG pathway enrichment displaying associations with Toll-like receptor signaling, complement cascades, ribosome, and DNA replication pathways.

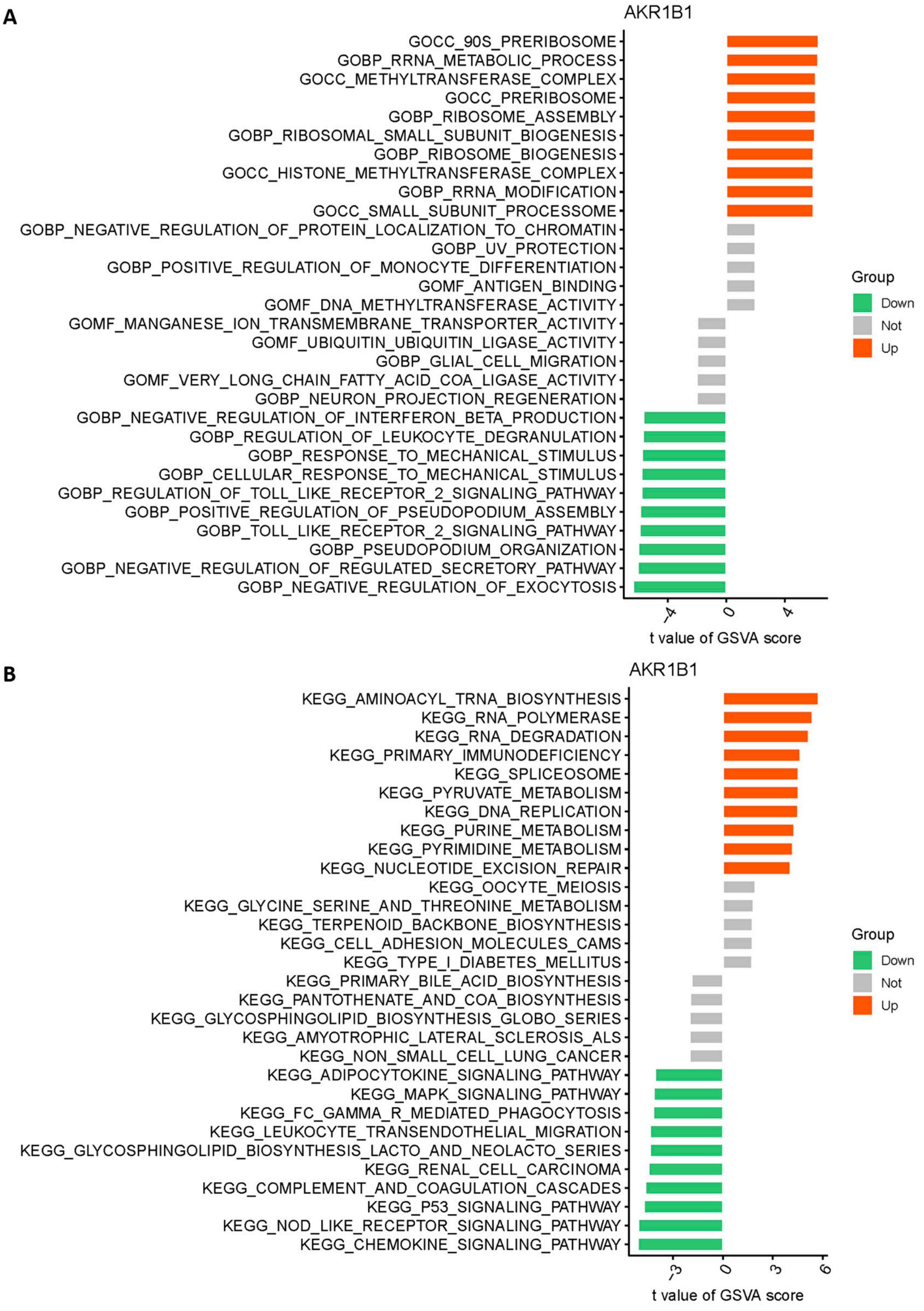

Figure S21. GSVA-GO and KEGG correlation bar plots for *AKR1B1*. (A) *AKR1B1* positively correlated with ribosome biogenesis- and rRNA metabolism-related GO terms, whereas negatively correlated terms were mainly associated with interferon-β regulation, leukocyte degranulation, and Toll-like receptor 2 signaling. **(B)** KEGG analysis showed positive correlations with aminoacyl-tRNA biosynthesis, RNA polymerase, RNA degradation, and nucleotide metabolism, while immune- and inflammation-related pathways, including

chemokine signaling, NOD-like receptor signaling, and leukocyte transendothelial migration, were negatively correlated.

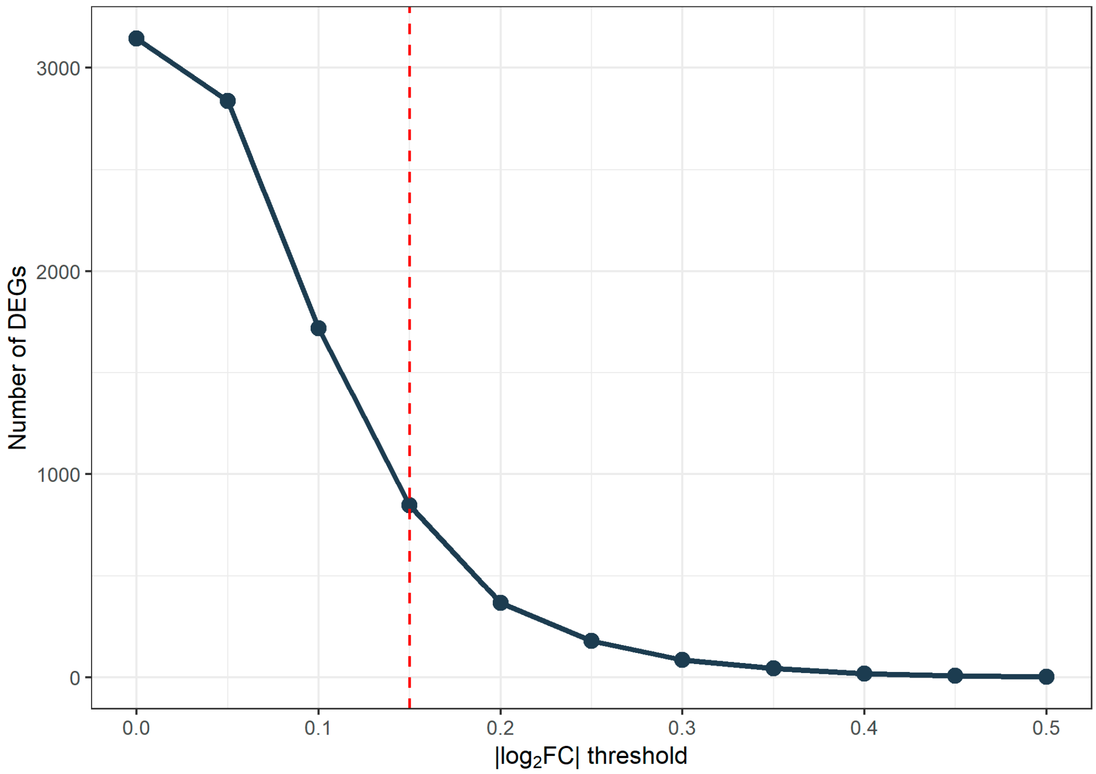

Figure S22. Sensitivity analysis of differentially expressed gene (DEG) identification across varying |log2FC| thresholds. The number of DEGs decreased markedly with increasing |log2FC| thresholds. The red dashed line marks the threshold adopted in the main analysis (|log2FC| = 0.15), indicating that the selected cutoff retained substantial differential signals while avoiding overly permissive DEG calling.
