# Supplementary material for: Multi-Omics Integration Identifies a Six-Gene Diagnostic Signature for Ankylosing Spondylitis via Metabolic–Immune Crosstalk
Source: Int J Mol Sci. 2026 Apr 27;27(9):3860. doi: 10.3390/ijms27093860 (PMC13164271; doi:10.3390/ijms27093860)
Supplement: Supplementary file 1 [file ijms-27-03860-s001.zip › Supplementary_Table_S1.pdf]

Table S1. Characteristics of the 16 metabolism-related candidate genes identified by WGCNA-DEG intersection and GeneCards filtering.

X	logFC	P.Value	adj.P.Val	Direction	absFC	IsHub
APOBEC3A	-0.3036575	5.23e-03	5.01e-02	Downregulated	0.3036575	No
NRD1	-0.2303515	2.00e-06	5.08e-04	Downregulated	0.2303515	No
AKR1B1	0.2282018	1.90e-05	1.82e-03	Upregulated	0.2282018	Yes
MFN2	-0.2054039	1.48e-03	2.37e-02	Downregulated	0.2054039	Yes
TLK1	-0.1972982	3.44e-04	9.81e-03	Downregulated	0.1972982	No
SLC27A3	0.1773904	6.43e-04	1.44e-02	Upregulated	0.1773904	Yes
MFSD1	-0.1761565	8.16e-03	6.50e-02	Downregulated	0.1761565	No
FASN	0.1728022	3.23e-04	9.33e-03	Upregulated	0.1728022	No
NCKAP1L	0.1673187	2.88e-04	8.80e-03	Upregulated	0.1673187	No
EXOSC10	0.1660610	6.61e-06	1.03e-03	Upregulated	0.1660610	No
UBE4A	-0.1659070	9.75e-06	1.36e-03	Downregulated	0.1659070	No
ZNF428	0.1642095	1.40e-03	2.31e-02	Upregulated	0.1642095	No
MTPN	-0.1639940	1.39e-04	5.58e-03	Downregulated	0.1639940	No
LCOR	-0.1627795	2.72e-05	2.12e-03	Downregulated	0.1627795	Yes
RHOB	-0.1583104	2.65e-02	1.32e-01	Downregulated	0.1583104	Yes
SMG7	-0.1508393	1.31e-05	1.48e-03	Downregulated	0.1508393	Yes
